# Copy number alteration features in pan-cancer homologous recombination deficiency prediction and biology

**DOI:** 10.1038/s42003-023-04901-3

**Published:** 2023-05-16

**Authors:** Huizi Yao, Huimin Li, Jinyu Wang, Tao Wu, Wei Ning, Kaixuan Diao, Chenxu Wu, Guangshuai Wang, Ziyu Tao, Xiangyu Zhao, Jing Chen, Xiaoqin Sun, Xue-Song Liu

**Affiliations:** 1 School of Life Science and Technology, ShanghaiTech University, Shanghai, China; 2 Shanghai Institute of Biochemistry and Cell Biology, Chinese Academy of Sciences, Shanghai, China; 3 University of Chinese Academy of Sciences, Beijing, China; 4 Shanghai Clinical Research and Trial Center, Shanghai, China

**Keywords:** Tumour biomarkers, Cancer screening, Cancer genomics

## Abstract

Homologous recombination deficiency (HRD) renders cancer cells vulnerable to unrepaired double-strand breaks and is an important therapeutic target as exemplified by the clinical efficacy of poly ADP-ribose polymerase (PARP) inhibitors as well as the platinum chemotherapy drugs applied to HRD patients. However, it remains a challenge to predict HRD status precisely and economically. Copy number alteration (CNA), as a pervasive trait of human cancers, can be extracted from a variety of data sources, including whole genome sequencing (WGS), SNP array, and panel sequencing, and thus can be easily applied clinically. Here we systematically evaluate the predictive performance of various CNA features and signatures in HRD prediction and build a gradient boosting machine model (HRD_CNA_) for pan-cancer HRD prediction based on these CNA features. CNA features BP10MB[1] (The number of breakpoints per 10MB of DNA is 1) and SS[ > 7 & <=8] (The log10-based size of segments is greater than 7 and less than or equal to 8) are identified as the most important features in HRD prediction. HRD_CNA_ suggests the biallelic inactivation of *BRCA1*, *BRCA2*, *PALB2*, *RAD51C*, *RAD51D*, and *BARD1* as the major genetic basis for human HRD, and may also be applied to effectively validate the pathogenicity of BRCA1/2 variants of uncertain significance (VUS). Together, this study provides a robust tool for cost-effective HRD prediction and also demonstrates the applicability of CNA features and signatures in cancer precision medicine.

## Abstract (web summary)

Analysis of copy number patterns of cancer genomes helps in devising a machine learning algorithm that can accurately predict homologous recombination deficiency status.

## Introduction

DNA damage is a common hallmark of cancer, and DNA double-strand break (DSB) is the most hazardous type of DNA damage which can be repaired by error-free homologous recombination (HR) pathway^1^. In cancer, accurate detection of homologous recombination deficiency (HRD) is of clinical relevance as HRD tumors are reported to be sensitive to poly ADP-ribose polymerase (PARP) inhibitors^2^, as well as to platinum chemotherapy drugs^3^.

Germline BRCA genes testing^4^ and the genomic instability score (GIS) are the currently widely used HR status diagnostic schemes. Germline BRCA testing could overlook the epigenetic silencing^5–8^, and other pathogenic somatic mutations^9^, and at the same time strongly relies on the completeness and accuracy of clinical variant annotation databases. GIS, also named as “HRD score”, mainly consists of three SNP array-based methods: telomeric allelic imbalance (TAI)^10^, loss of heterozygosity (LOH)^11^, and large-scale transition (LST)^12^. One disadvantage of HRD score is that the optimal threshold is not consistent in patients with different types of cancer^13–15^. Recently, HRDetect^16,17^ and CHORD^9^ have been constructed for HRD prediction, however, these tools need whole genome sequencing (WGS) or whole exome sequencing (WES) data, which is expensive and lacks clinical applicability.

Copy number alteration (CNA) is an important type of cancer-driving genetic alteration, and CNA signatures and features are emerging cancer-inherent patterns. Recently, patterns and mutational processes of CNA signature have started to be revealed^18–21^. CNA information can be obtained from a diverse type of data, such as shallow WGS, WES, SNP array, and panel sequencing, and could represent a cost-effective type of biomarker for cancer diagnosis and clinical response prediction. However, the efficacy and application of CNA signatures and features in HRD prediction have not been established yet.

Here we develop a robust HRD predictor HRD_CNA_ (Homologous recombination deficiency prediction by copy number alteration features) based on CNA features. HRD_CNA_ model is trained on the CNA data of 1,470 cancer samples generated from WGS or SNP array and is validated with 831 samples generated from WGS, SNP array, shallow WGS, or panel sequencing data. HRD_CNA_ can precisely predict HR status using CNA features data derived from different platforms or different cancer types with consistent cut-off. HRD_CNA_ provides a new direction for effectively validating the pathogenicity of uncertain important variants (VUS) of BRCA1 and BRCA2, and suggests the major genetic basis for human HRD. The applicability of CNA features and signatures in cancer precision medicine is also well demonstrated in HRD_CNA_.

## Results

### Data collection for HRD prediction model training and validation

**Fig. 1:**
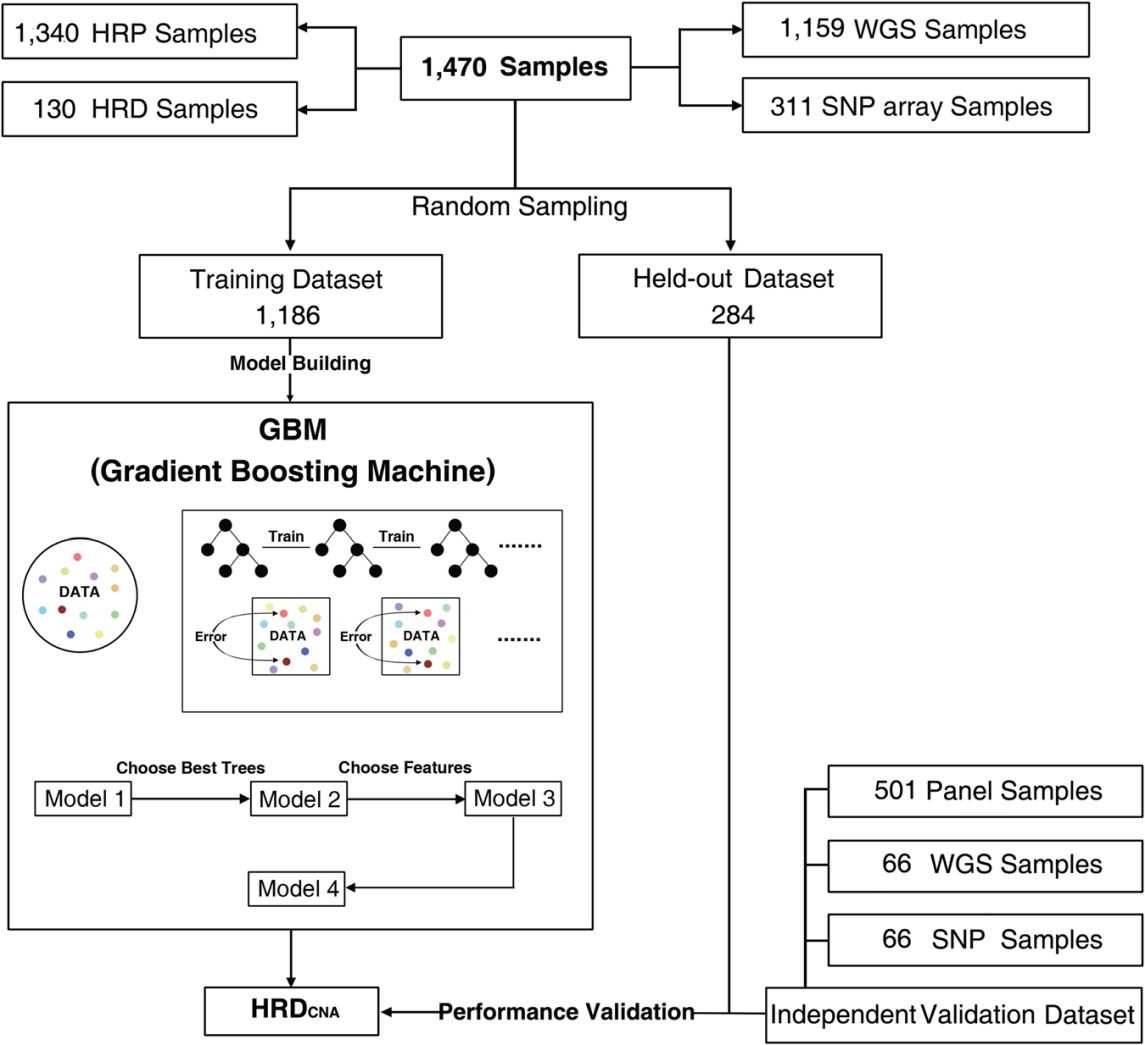
HRD_CNA_ is a gradient boosting machine for pan-cancer homologous recombination deficiency (HRD) prediction. Training and validation processes of HRD_CNA_, which outputs the probability of being HRD.

For the development of the HRD prediction model, 1854 samples (WGS data) from the pan-cancer analysis of whole genomes^9^ (PCAWG) and 560 breast cancer samples (SNP array data)^16^ are collected. To obtain a high-confidence training dataset of HRD, samples with BRCA1/2 deficiency are screened for classifier training. In total, 130 cancer samples with loss-of-function mutations in BRCA1/2 are labeled as HRD, while 1340 samples without inactivation mutations in known HR genes are labeled as homologous recombination proficiency (HRP). Then these samples are randomly split into training and held-out datasets by the ratio of 8:2 for model training and testing respectively. The performance of the HRD prediction model is further evaluated on three independent validation datasets: a total of 633 cancer samples, including 66 breast cancer samples with WGS, 66 breast cancer samples with SNP array sequencing, and 501 pan-cancer samples with panel sequencing (Fig. 1).

### Selection of the method for HRD prediction model building

To find the best HRD prediction model, a total of 9 machine learning models (see Methods) are trained using the training dataset, and the performances of each machine learning model are reported as the area under the receiver operating characteristic (ROC) curve (AUC) and the area under the precision-recall curves (PR-AUC) in all datasets (Supplementary Fig. 1a–f). Gradient boosting machine (GBM) model shows the best performance in AUC and other metrics like accuracy, precision, and F1 score on the held-out dataset (Supplementary Fig. 1g, h), thus it is selected for subsequent HRD prediction model development (Fig. 1).

### HRD_CNA_, pan-cancer HRD predictor based on CNA features

**Fig. 2:**
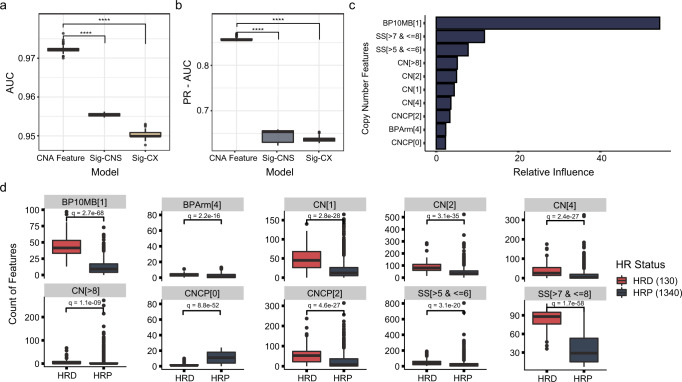
HRD_CNA_, choosing CNA features rather than CNA signatures to predict HRD. **a**, **b** Performance comparisons between the HRD prediction models constructed using CNA features, or the activities of two sets of CNA signatures, Sig-CNS (Method from Wang et al.) or Sig-CX (Method from Ruben et al.). The differences in the area under the receiver operating characteristic curves (AUC) **a** and the area under the precision-recall curves (PR-AUC) **b** on the held-out dataset are shown. ****P < 0.0001. *P* values are calculated using Wilcoxon test. **c** The features used by HRD_CNA_ to predict HRD and their contributions. **d** The difference in 10 CNA features between HRD and HRP samples. *Q* values are calculated using Wilcoxon test.

CNA signatures and features are emerging cancer inherent indicators. Wang et al.^20^. classified CNA segments based on 8 types of CNA features, then performed the first CNA signature analysis in prostate cancer using the non-negative matrix factorization (NMF) algorithm with these CNA features, and the CNA signatures developed with this method are named “Sig-CNS” here^20^. Ruben et al. conducted a pan-cancer CNA signature study and reported 17 signatures (named “Sig-CX” here)^19^. CNA features are countable parameters developed in Wang et al. study^20^. We compare the performance of three different models using CNA features or using the reported two sets of CNA signatures (Sig-CNS and Sig-CX). AUC and PR-AUC results show that HRD prediction models construct with CNA features exhibit improved performance compared with models built with CNA signatures (Fig. 2a, b), thus CNA features are chosen for subsequent HRD model development.

To ensure that the parameters identified are robust and generalizable, 10-fold cross-validation (CV) strategy is used. Then the relative variable importance is calculated^22^, and the features with the top 10 relative influence score among all features in the model are selected as the optimal modeling features (Fig. 2c). Then we select the cancer types with higher HRD incidence to analyze the differences in the 10 features (Supplementary Fig. 2). The analysis results show that these features have significant differences in HRD and HRP samples, not only in pan-cancer but also in individual cancers. Finally, we trained an HRD model using these selected CNA features (Fig. 2d). The BP10MB[1] and SS[ > 7 & <=8] are found to be the top two important predicting factors of HRD (Fig. 2c). The final model is named “HRD_CNA_”.

### HRD_CNA_ model validation and application

**Fig. 3:**
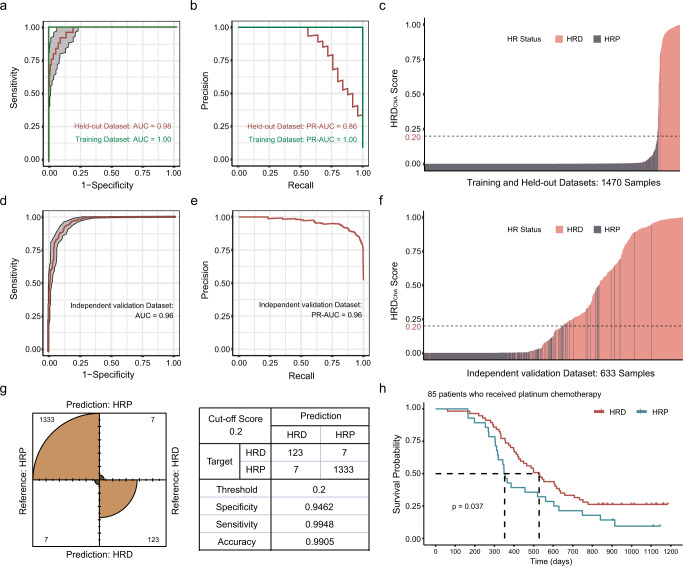
Performance of HRD_CNA_ model. **a**, **b** ROC curves **a** and PR curves **b** analysis showed the performance of HRD_CNA_ built using CNA features on the training and held-out datasets. The gray shaded area represents a 95% confidence interval. **c** HRD_CNA_ scores for 1470 pan-cancer samples in training and held-out datasets are ordered from lowest to highest. The HRD_CNA_ scores of almost all HRD samples are above 0.2. **d**, **e** The ROC curve **d** and PR curve **e** showed the performance of HRD_CNA_ model in the independent validation dataset of 633 cancer samples. The gray shaded area represents a 95% confidence interval. **f** HRD_CNA_ scores for 633 pan-cancer samples in three independent validation datasets are ordered from lowest to highest. The horizontal dashed line shows a cut-off score of 0.2. **g** The performance of HRD_CNA_ model in training and held-out datasets is visualized with a confusion matrix under cut-off score of 0.2. **h** Kaplan-Meier (KM) survival analysis. Patients for analysis included 85 patients who had received platinum chemotherapy from 501 pan-cancer cohort. Samples are grouped based on HRD_CNA_ scores. Patients with high HRD_CNA_ scores showed significantly improved survival when treated with platinum chemotherapy. *P* value is calculated using log-rank test.

HRD_CNA_ shows excellent performance in the held-out dataset and also three independent validation datasets (Fig. 3a–f). The performance of HRD_CNA_ model is not influenced by the CNA assay platforms (Supplementary Fig. 3). ShallowHRD, a tool to evaluate tumor HRD based on a CNA profile derived from WGS at low sequencing depth^23^, shows 0.88 sensitivity and 0.91 specificity for HRD detection. Compared with ShallowHRD, HRD_CNA_ shows consistently improved performance using CNA profiles derived from different sequencing depths of WGS using downsampling (Supplementary Fig. 4). Since HRD cases disproportionately represented in some cancers, it remains to be verified whether HRD_CNA_ model applies to all cancer types. We verified the performance of HRD_CNA_ model in individual cancers separately. The results show that the model had excellent applicability in breast, ovarian, pancreatic lymphatic, prostate, and liver cancers (Supplementary Fig. 5).

We compare the performance of HRD_CNA_ with HRDetect, LOH, TAI, LST, and HRD score. HRD_CNA_ shows improved performance compared with HRDetect in HRD prediction in an independent cohort of 71 TNBC patients (Supplementary Fig. 6a). The scatterplot displays the relationship between HRD_CNA_ score and HRDetect score (Supplementary Fig. 6b). Since HRD score is not applicable to panel sequencing data, we use data from WGS and SPN array sequencing to compare its performance with HRD_CNA_, including 66 breast cancer WGS and SNP array samples. Compared with LST and HRD score, HRD_CNA_ has similar performance but is better than TAI and LOH (Supplementary Fig. 6c, d).

When evaluating the performance of the model, it is found that HRD_CNA_ scores of HRD samples are almost above 0.2, and HRP samples mostly get lower scores (Fig. 3c, f). Consequently, 0.2 was chosen as the threshold for HRD prediction (Fig. 3g). To further validate the performance of our model, we perform a Kaplan-Meier (KM) survival analysis on patients including 85 patients who had received platinum chemotherapy from 501 pan-cancer cohort^24^. Compared with patients with low HRD_CNA_ scores, patients with high HRD_CNA_ scores show significantly improved survival when treated with platinum chemotherapy (Fig. 3h). Then we compare our model with HRD score, a widely used HRD predicting method, and we define samples with HRD score ≥ 42 as HRD according to previous study^25^ (Supplementary Fig. 7). Survival analysis suggests that HRD_CNA_ shows similar performance as HRD score.

### BP10MB[1] and SS[ > 7 & <=8] as potential biomarkers of HRD

**Fig. 4:**
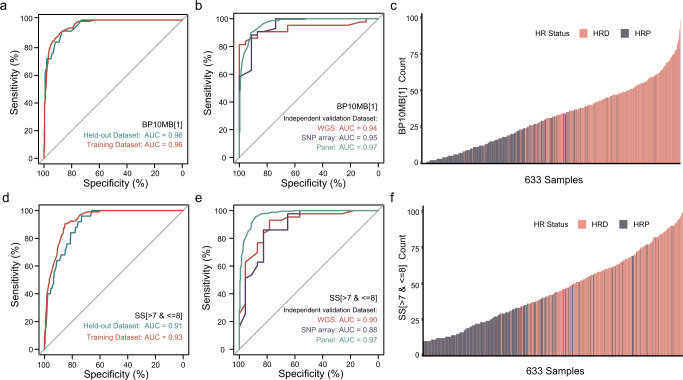
BP10MB[1] and SS[ > 7 & <=8] as potential biomarkers of HRD. **a** HRD prediction ROC curves of CNA feature BP10MB[1] in training and held-out datasets. **b** ROC curves showed the performance of CNA feature BP10MB[1] in three independent validation datasets with different sequencing platforms, a total of 633 cancer samples, including 66 breast cancer samples with WGS sequencing, 66 breast cancer samples with SNP array sequencing, and 501 pan-cancer cancer samples with panel sequencing. **c** BP10MB[1] count for 633 cancer samples is ordered from lowest to highest. **d** ROC curves of CNA feature SS[ > 7 & <=8] in training and held-out datasets. **e** ROC curves showed the performance of CNA feature SS[ > 7 & <=8] in three independent validation datasets. **f** SS[ > 7 & <=8] count for 633 cancer samples is ordered from lowest to highest.

The counts of CNA features significantly differ between HRD and HRP samples (Fig. 2d). Among these 10 selected CNA features for HRD_CNA_ model construction, the top two important components are BP10MB[1] and SS[ > 7 & <=8], and either feature could predict HRD with comparable performance to the HRD_CNA_ model (Fig. 4a, d, Supplementary Fig. 8). BP10MB[1] indicates the number of breakpoints per 10MB of DNA is 1 and SS[ > 7 & <=8] indicates the log10-based size of segments is greater than 7 and less than or equal to 8. The performance of BP10MB[1] and SS[ > 7 & <=8] is further verified with independent validation datasets (Fig. 4b, e). The counts of BP10MB[1] and SS[ > 7 & <=8] are enriched in HRD samples compared with HRP samples (Fig. 4c, f). Altogether, these results suggest that these two features BP10MB[1] and SS[ > 7 & <=8] may be potential biomarkers of HRD. Then we compare BP10MB[1]‘s performance in HRD prediction with LOH, TAI, and LST in a cohort of 80 new breast cancers^16^ (Supplementary Fig. 9a). BP10MB[1] perform better than LOH and TAI, but similar to LST. LST, defined as chromosomal breaks between adjacent regions of at least 10 MB or larger, is also considered a robust indicator of HR status^12^, the difference between LST and BP10MB[1] is shown (Supplementary Fig. 10). And BP10MB[1] also perform well in individual cancer types (Supplementary Fig. 9b).

### Genetic basis for human HRD

**Fig. 5:**
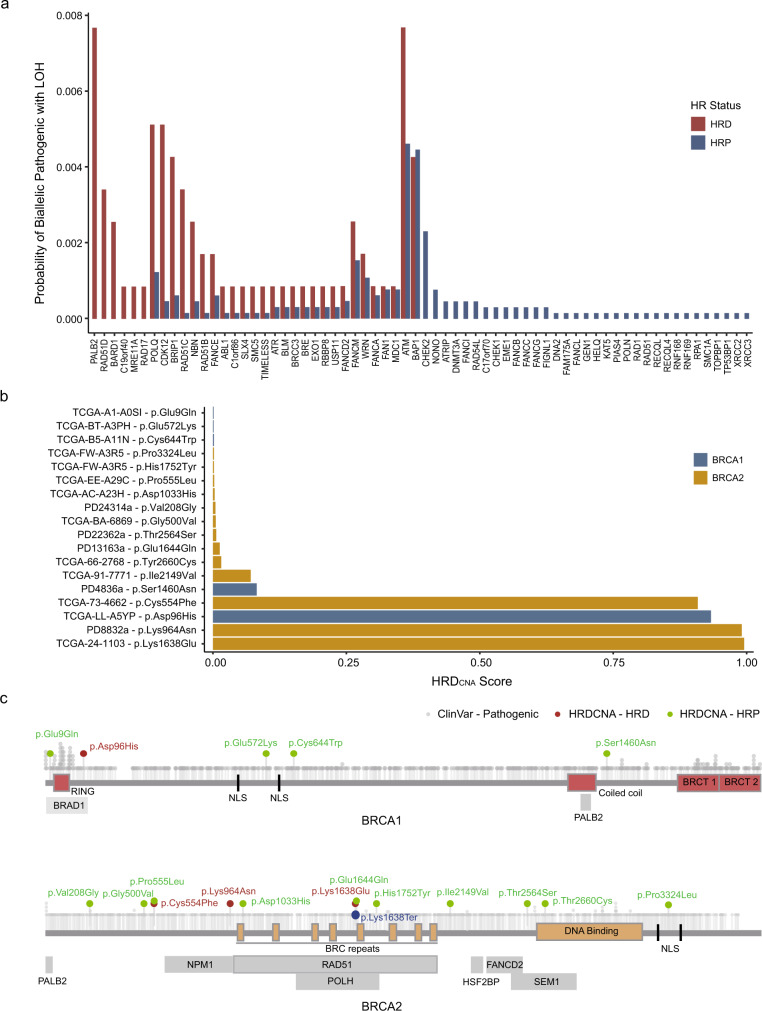
Genetic basis for HRD and BRCA VUS reclassification. **a** The incidence of biallelic pathogenic mutations in HR-related genes in samples that are predicted to be HRD or HRP by HRD_CNA_ in the TCGA dataset. **b** The HRD_CNA_ scores for TCGA and 560 breast datasets of cancer patients with BRCA1/2 VUS and LOH are shown. **c** Distributions of VUS on BRCA proteins. BRCA1/2 amino acid loci of known pathogenic variants and pathogenic variants predicted by HRD_CNA_ are shown. The position of functional regions of BRCA 1/2 proteins and interacting proteins are obtained from UniPort databases, and pathogenic variants are defined based on ClinVar database. Vertical positions represent different kinds of amino acids. BARD1 BRCA1-associated RING domain protein 1, NLS nuclear localization sequence, PALB2 partner and localizer of BRCA2, BRCT BRCA1 C-terminus, BRC repeats ~30–40 residue long sequence regions in BRC2 protein, NPM1 nucleophosmin 1, RAD51 DNA repair protein RAD51 homolog 1, POLH DNA Polymerase Eta, HSF2BP Heat shock factor 2-binding protein, FACD2 Fanconi anemia group D2 protein, SEM1 26 S proteasome complex subunit SEM1.

BRCAness is functionally defined as a defect in homologous recombination repair that phenocopies loss of BRCA1/2^26^. In addition to BRCA1/2, pathogenic variants in BRCAness genes such as partner and localizer of BRCA2 (*PALB2*) have been reported to contribute to human HRD^9,27^. We explore potential BRCAness genes using the cancer genome atlas (TCGA) dataset by comparing the incidence of biallelic pathogenic mutations in HR-related genes^28^ between patients predicted as HRD and HRP using HRD_CNA_ (Fig. 5a). The incidence of biallelic pathogenic mutations in *PALB2* and RAD51 paralog C (*RAD51C*) is observed to be significantly higher in HRD, and the inactivation of these two genes have been demonstrated in previous studies as the major genetic basis of human HRD^29–31^. Patients with biallelic inactivation mutations in the following genes: RAD51 paralog D (*RAD51D*) (*n* = 4), BRCA1-associated RING domain protein 1 (*BARD1*) (*n* = 3), Chromosome 19 open reading frame 40 (*C19orf40*) (*n* = 1) and MRE11 homolog A (*MRE11A*) (*n* = 1) are all predicted as HRD, suggesting these genes, especially *BARD1* and *RAD51D* inactivation can be genetic basis of human HRD.

HRD_CNA_ is applied to 33 cancers in the TCGA dataset, each sample gets an HRD_CNA_ score. The scores widely vary across different cancer types. The highest scores are observed in ovarian cancer, and many cancer types also have subsets of samples with high HRD_CNA_ scores. Some cancer types such as thyroid carcinoma, thymoma, uveal melanoma, and acute myeloid leukemia do not show high HRD_CNA_ scores (Supplementary Fig. 11). Similar pan-cancer HRD distribution patterns have been reported previously^9^.

### BRCA VUS classification

The majority of missense mutations in BRCA1/2 have been classified as variants of uncertain significance (VUS) with unknown functional consequences. We calculate the HRD_CNA_ scores of 11,465 samples from 560 breast and TCGA datasets. Samples with BRCA VUS mutations and LOH in BRCA alleles are collected, and samples with known pathogenic HRD-causing genetic alterations are removed. HRD_CNA_ classified variants with scores above 0.2 as “likely pathogenic” and those with scores below 0.2 as “likely benign”. A total of 18 samples with homozygous BRCA VUS mutations are found, and 4 of them are classified as HRD based on HRD_CNA_ scores (Fig. 5b). Then we include the labeling information from other tools such as Cancervar, CADD, etc. for the ClinVar VUS clarified with HRD_CNA_ (Supplementary Table 1). Pathogenic BRCA1/2 missense mutations in ClinVar are displayed (Fig. 5c), and functional domains are enriched with these pathogenic mutations^32,33^. 5 variants with high HRD_CNA_ scores are mainly distributed in the domains of BRCA1/2 proteins that interact with other proteins. For example, BRCA1 p.Asp96His is situated in the domain responsible for the interaction between BRCA1 and BARD1.

## Discussion

Here we developed a pan-cancer HRD prediction tool HRD_CNA_ based on CNA features. This tool enables accurate and robust HRD prediction using CNA profiles derived from a variety of cost-effective platforms, such as shallow WGS, SNP array, and panel sequencing. Furthermore, the CNA features BP10MB[1] and SS[ > 7 & <=8] are identified as the major contributors to HRD_CNA_ model, and also represent potential biomarkers for HRD. In addition to biallelic inactivation of *BRCA1* and *BRCA2*, *PALB2*, *RAD51C*, *RAD51D*, and *BARD1* inactivation are identified as the major genetic basis for human HRD. HRD_CNA_ tool also provides a new direction for effectively validating the pathogenicity of VUS. Carrying BRCA1/2 mutations, earlier stage, and lower levels of residual tumor after surgery have been proven as predictors of better prognosis for patients receiving platinum chemotherapy in ovarian cancer^34,35^. Here, survival analysis shows that patients with high HRD_CNA_ scores have significantly improved survival than those with low HRD_CNA_ scores in patients receiving platinum chemotherapy. Over time, the application of HRD_CNA_ would unearth a substantial cohort of patients without BRCA1/2 mutations for chemotherapy using platinum drugs.

In addition to *BRCA1* and *BRCA2*, *PALB2* and *RAD51C* biallelic pathogenic mutations have been demonstrated in previous studies as the major genetic basis of human HRD^29–31^. Notably, there is also a relatively high incidence of biallelic alterations in *BARD1* and *RAD51D* in HRD. BRCA1 partners with BARD1 to mediate the initial nucleotide excision of DNA damage and the recruitment of the recombinase RAD51^36^. *BARD1* loss is sufficient to confer an HRD phenotype and significantly increase sensitivity to PARP inhibitors in prostate cancer cells^37^. Triple-negative breast cancer cells carrying *RAD51D* K91fs and V200X variants are reported to be vulnerable to PARP inhibitors because they lead to decreased HR function^38^. Together, our study suggests *BRCA1*, *BRCA2*, *PALB2*, *RAD51C, BARD1*, and *RAD51D* biallelic inactivation as the major genetic basis of human HRD, and it provides a novel opportunity for the precision medicine approaches in HRD and expands the population of patients who may benefit from agents targeting HRD.

Collectively, accurate measurement of HR status to find HRD patients has great clinical significance in precision medicine. HRD_CNA_ model is a CNA features-based robust tool for HRD detection. In the future, CNA features or signatures could be combined with artificial intelligence to reveal clinically meaningful CNA fingerprints for cancer precision diagnosis and therapy response prediction, and the HRD_CNA_ model developed in this study could represent one example of these clinically meaningful CNA fingerprints. HRD_CNA_ is freely available as an R package at https://github.com/XSLiuLab/HRDCNA.

## Methods

### Datasets

We have used CNA data from 1470 samples to develop the homologous recombination deficiency (HRD) gradient boosting machine (GBM) model, called HRD_CNA_ (Homologous recombination deficiency prediction by copy number alteration features), which included 1159 samples (WGS data) from PCAWG dataset^9^, 311 samples (SNP array data) from 560 breast dataset^16^. In particular, samples that were duplicated with PCAWG dataset in 560 breast dataset have been excluded, so there are no duplicated samples in our datasets. The information about the frequencies of cancer types in the training data for HRD_CNA_ model is shown in Supplementary Table 2. Somatic copy number data for the international cancer genome consortium (ICGC) portion of PCAWG dataset is downloaded at https://dcc.icgc.org/releases/PCAWG/. BRCA1/2 status annotations for this dataset are obtained from the supplementary data in Nguyen et al.^9^. Somatic copy number data of the 560 breast dataset are downloaded from the department of medical genetics at the University of Cambridge (http://medgen.medschl.cam.ac.uk/serena-nik-zainal/). BRCA1/2 status annotations and mutation data for this dataset are obtained from the supplementary data in Davies et al.^16^.

130 samples carrying mutations in BRCA1/2 are detected with loss of wild-type allele and labeled as HRD samples, and 1340 BRCA1/2 proficiency samples as HRP samples. A sample with one of the following events in BRCA1/2 is defined as a BRCA1/2 deficient sample: (i) complete copy number loss, (ii) LOH in combination with a pathogenic germline or somatic SNV/indel or structural variations, or (iii) pathogenic SNV/indels or structural variations in both alleles^9^. We select 80% of 1470 samples by random sampling as the training dataset, and 20% as the held-out dataset for model training. Copy number data, HR status information, and survival data for the panel dataset are obtained from the supplementary data in Wen H et al.^24^. 66 breast dataset is publicly available in the figshare repository provided by de Luca et al.^39^. Copy number data from ASCAT (array data) are available from 10.6084/m9.figshare.9808496 and ascatNGS (for original (10.6084/m9.figshare.9808505) and downsampled WGS data are available from (30×: 10.6084/m9.figshare.9808511, 15×: 10.6084/m9.figshare.9808514, 10×: 10.6084/m9.figshare.9808517)).

Somatic copy number data of TCGA pan-cancer dataset are downloaded from genomic data commons data portal (https://portal.gdc.cancer.gov/). Allele-specific copy number analysis of tumors is performed using ASCAT2, to generate integral allele-specific copy number profiles for the tumor cells. Biallelic inactivation in HR-related genes information for TCGA dataset is obtained from Riaz et al.^28^. The mutation data for TCGA dataset are obtained from the GDC Data Portal and Legacy Archive (https://gdc.cancer.gov/). The information on the cohort of 71 TNBC patients in TCGA dataset is derived from the supplementary data in Liao et al.^40^. The information on the cohort of 80 breast patients is derived from the supplementary data in Davies et al.^16^. Pathogenicity annotations are obtained from ClinVar (https://www.ncbi.nlm.nih.gov/clinvar/), CancerVar^41^ (https://cancervar.wglab.org/), CADD^42^ (https://cadd.gs.washington.edu/), MutationAssessor^43^ (http://mutationassessor.org/r3/) and PolyPhen-2^44^ (http://genetics.bwh.harvard.edu/pph2/).

### Downsampling of WGS data

Downsampling of the original normal and tumor BAM files is performed using the samtools (version 1.3)^45^ library function: samtools view -h -s x, where x represents the desired percentage of downsampling required (The values we use that will result in an approximate coverage of 30×, 15×, and 10×, depending on the coverage of the original data).

### Construction of machine learning models

To find the best performance model among a multitude of methods, 9 machine learning models including extremely randomized trees (Extra trees), random forest, logistic regression, support vector machine (SVM), eXtreme gradient boosting (XGB), adaptive boosting (AdaBoost), decision tree, K-nearest neighbor (K-Neighbor) and gradient boosting machine (GBM) are trained, the AUC of the held-out dataset is selected as the performance criterion. All the hyper-parameters are determined by the gradient search method provided by the *GridSearchCV* function in Python package *sklearn*, and we use the default 5-fold CV strategy. The GBM model has the best performance on the held-out dataset. Thus, GBM is chosen to predict the probability of HRD. Gradient boosting is a machine learning technique for regression and classification problems, which produces a prediction model in the form of an ensemble of weak base models, usually decision tree. R package *gbm* is used to implementation of the GBM.

### Quantification of CNA signatures and CNA features

Sig-CNS is identified using R package *Sigminer*^20^, which is based on the tool *SigProfiler*^46^ caller. We pick up 8 signatures for our dataset due to its relatively high stability and low distance (Supplementary Fig. 12). Identification of Sig-CX used R package *CINSignatureQuantification*^19^. Calling CNA features is performed using R package *Sigminer*^20^. 8 fundamental CNA features are computed, including the breakpoint count per 10 Mb (named BP10MB); the breakpoint count per chromosome arm (named BPArm); the copy number of the segments (named CN); the difference in copy number between adjacent segments (named CNCP); the lengths of oscillating copy number segment chains (named OsCN); the log10 based size of segments (named SS); the minimal number of chromosome with 50% copy number variation (named NC50); the burden of chromosome (named BoChr). Then we classified 8 CNA feature distributions into 80 different components^20^, and each copy number component has a clear biological meaning, for example, BP10MB[1] indicates the number of breakpoints per 10MB of DNA is 1 (Details in Supplementary Table 3). Three models are built using CNA features directly or using two CNA signatures, Sig-CNS and Sig-CX, each of which is modeled repeatedly using Monte Carlo CV, which is repeated 100 times on the 80% dataset. The difference in AUC and PR-AUC of the three models is compared on the held-out dataset. CNA features are finally chosen for model development.

### HRD_CNA_ training procedure

The model training includes a 4-step training procedure to obtain the final model (named HRD_CNA_). Firstly, the HRD_CNA_ model is developed based on 76 significantly different CNA features among 80 CNA features (Supplementary Fig. 13) and the correlation matrix shows that no significant correlation is observed between most features (Supplementary Fig. 14). We choose bagging fraction 0.8 for more robust results. In addition, not to miss the optimal solution, we use a small learning rate of 0.01 together with a large number of trees (6000). We carry out 10-fold CV on the 80% training dataset and determine the best number of trees (572) from the Bernoulli deviance (Supplementary Fig. 15a). Then, from the relative influence score of features for the model, some CNA features are fewer contributions to our model, so we reduce model features. To calculate the contribution more accurately, we perform Monte Carlo CV on the training dataset using 76 CNA features, which are repeated 500 times. To calculate the relative influence of the specific variable, at each split in each tree, GBM computes the improvement in squared error, then averages the improvement made by each variable across all the trees that the variable is used^22^. We choose 10 CNA features with the top 10 relative influence scores among all features. Moreover, we again perform 10-fold CV on the training dataset using these 10 CNA features and determine the best number of trees (777) from the Bernoulli deviance (Supplementary Fig. 15b). Finally, we train our final model using 10 significantly different and important CNA features on the 80% training dataset.

### Statistic and reproducibility

Data between two groups are compared using Wilcoxon rank-sum test (also known as “Mann–Whitney” test) depending on the normality of data distribution. In the survival analysis, the differences between different survival curves are compared using log-rank test, and the cut-off score is 0.14, which is determined based on the youden index^47^. All reported *P*-values are two-tailed, and for all analyses, *P* ≤ 0.05 is considered statistically significant unless otherwise specified. All statistical analysis is performed using R v4.1.0. The area under the curves of the receiver operating characteristic curves (AUC) and the area under the precision-recall curves (PR-AUC) are then calculated by R package *precrec*.

CNA data to develop HRD_CNA_ includes 1470 samples with 1186 in the training dataset and 284 in the held-out dataset. 130 samples are labeled as HRD samples, and 1340 samples are labeled as HRP samples. The independent validation dataset consists of 633 cancer samples, including 66 breast cancer samples with WGS, 66 breast cancer samples with SNP array sequencing, and 501 pan-cancer samples with panel sequencing, and 330 of which are HRD samples and 303 HRP samples. TCGA dataset of 33 cancer types includes 10,906 samples. The cohort of 71 TNBC patients in TCGA dataset consists of 71 TNBC samples, 43 of which are HRD samples and 28 HRP samples. The cohort of 80 breast patients consists of 80 breast samples, 7 of which are HRD samples and 73 HRP samples.

### Reporting summary

Further information on research design is available in the Nature Portfolio Reporting Summary linked to this article.

## Supplementary information

Supplementary Information

Reporting Summary

## Data Availability

All data used in this study are obtained from publicly available sources and are described in detail in Supplementary Table 4. The datasets generated and analyzed during the current study are available at https://github.com/XSLiuLab/InterpretationAnalysisHRDCNA^48^. Any remaining information can be obtained from the corresponding author upon reasonable request.

## Code availability

HRD_CNA_ is freely available as an R package at https://github.com/XSLiuLab/HRDCNA. The code used for data processing and generating the figures to reproduce the main results of this work is also publicly accessible at https://github.com/XSLiuLab/InterpretationAnalysisHRDCNA^48^.
