# Supplementary material for: Copy number alteration features in pan-cancer homologous recombination deficiency prediction and biology
Source: Commun Biol. 2023 May 16;6:527. doi: 10.1038/s42003-023-04901-3 (PMC10188435; doi:10.1038/s42003-023-04901-3)
Supplement: Supplementary file 1 — Supplementary Information [file 42003_2023_4901_MOESM1_ESM.pdf]

# Supplementary Figure 1

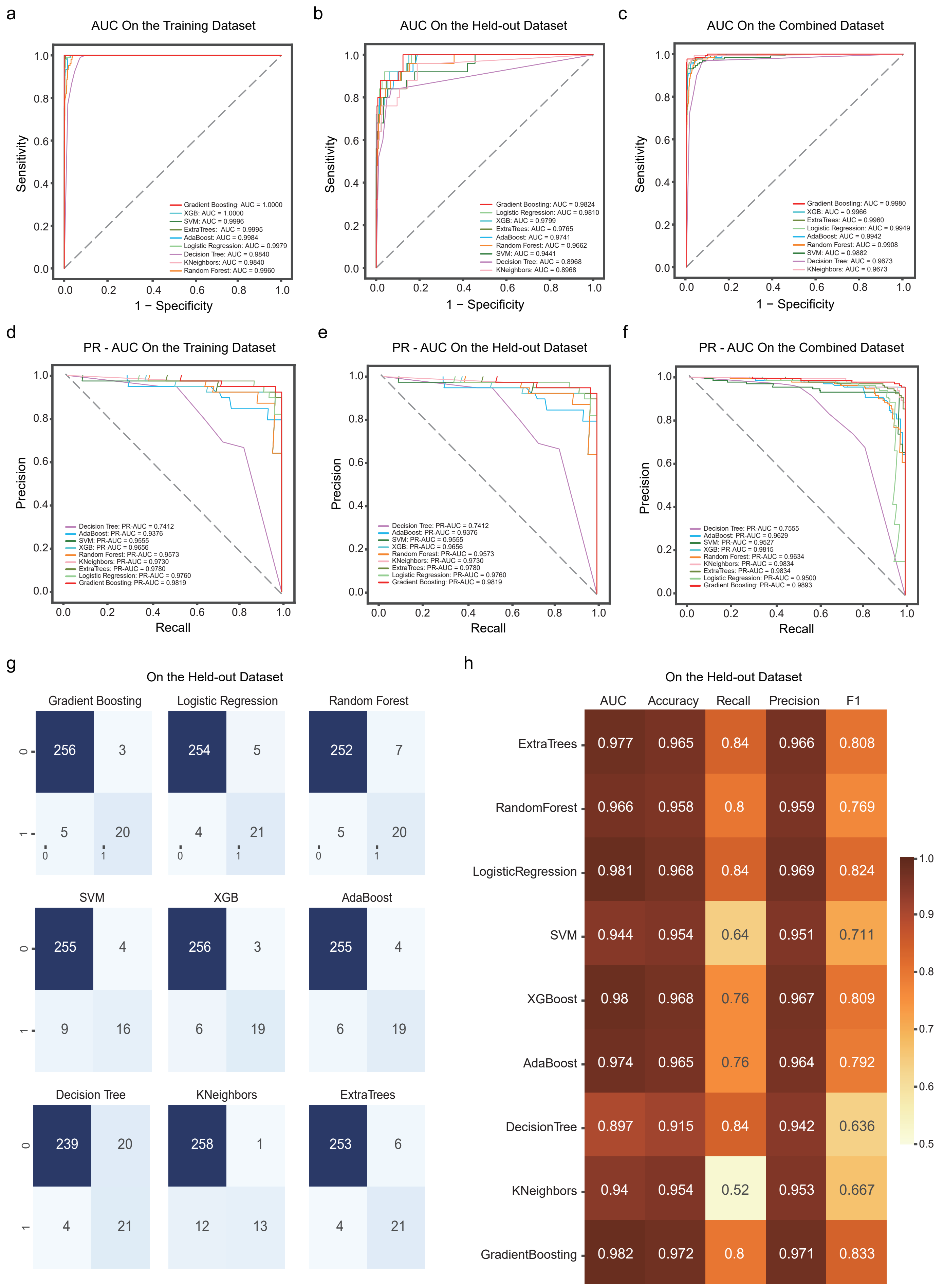

**Supplementary Fig. 1: Selection of the method for HRD prediction model building.**

GBM shows the best performance among the 9 machine learning methods. **a-c** AUC of 9 models on training (**a**), held-out (**b**), and combined (**c**) datasets. **d-f** PR-AUC of 9 models on training (**d**), held-out (**e**), and combined (**f**) datasets. **g** Confusion matrix of 9 machine learning models on the held-out dataset, including extremely randomized trees (Extra Trees), random forest, logistic regression, support vector machine (SVM), extreme gradient boosting (XGB), adaptive boosting (AdaBoost), decision tree, K-nearest neighbor (KNeighbor) and gradient boosting machine (GBM). **h** AUC, accuracy, recall, precision, and F1 score of 9 models on the held-out dataset are shown.

# Supplementary Figure 2

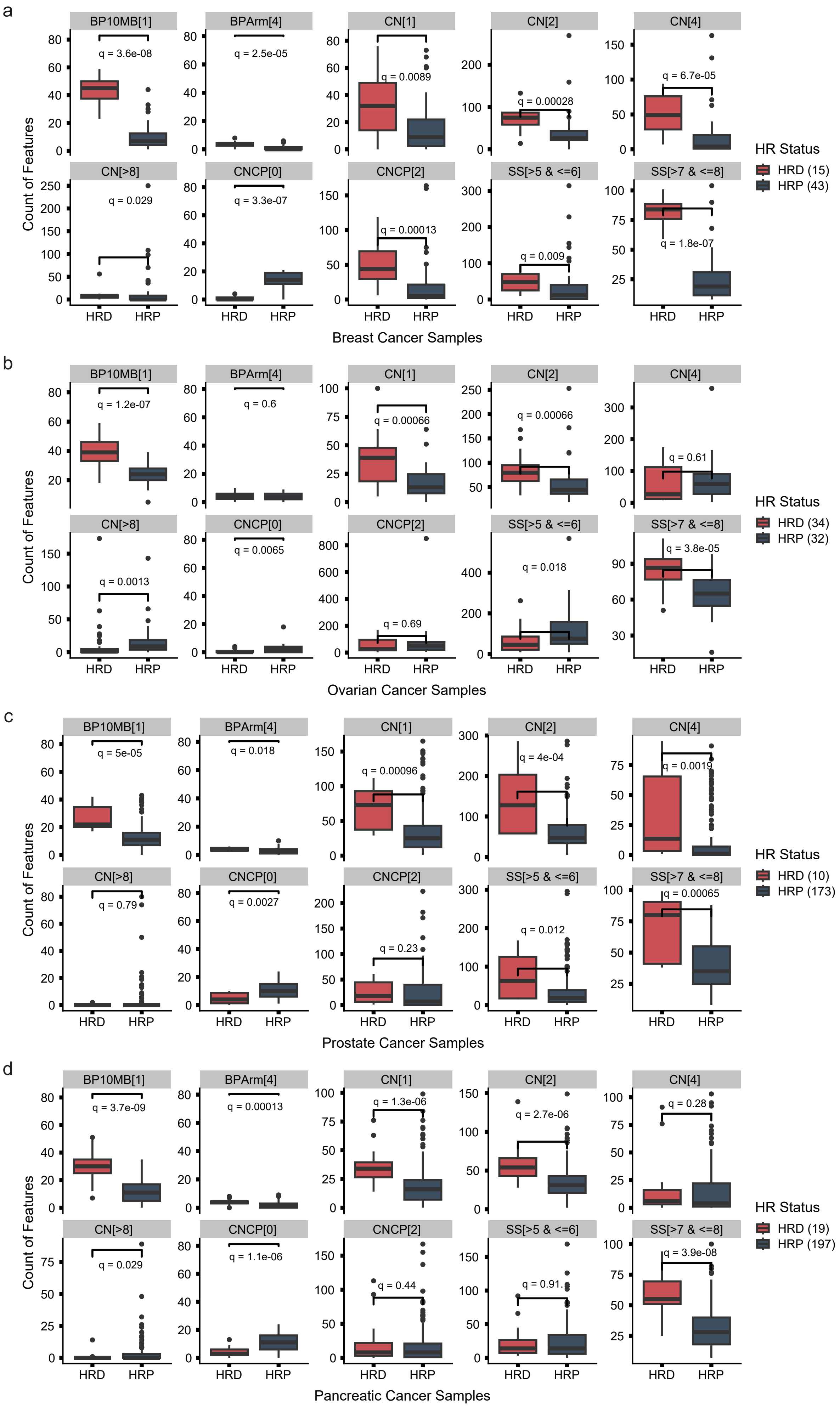

**Supplementary Fig. 2: The difference in 10 CNA features between HRD and HRP samples in individual cancer types.**

**a-d** The difference in 10 CNA features between HRD and HRP samples in breast (**a**), ovarian (**b**), prostate (**c**), and pancreatic (**d**) cancer. *Q* values are calculated using Wilcoxon test.

# Supplementary Figure 3

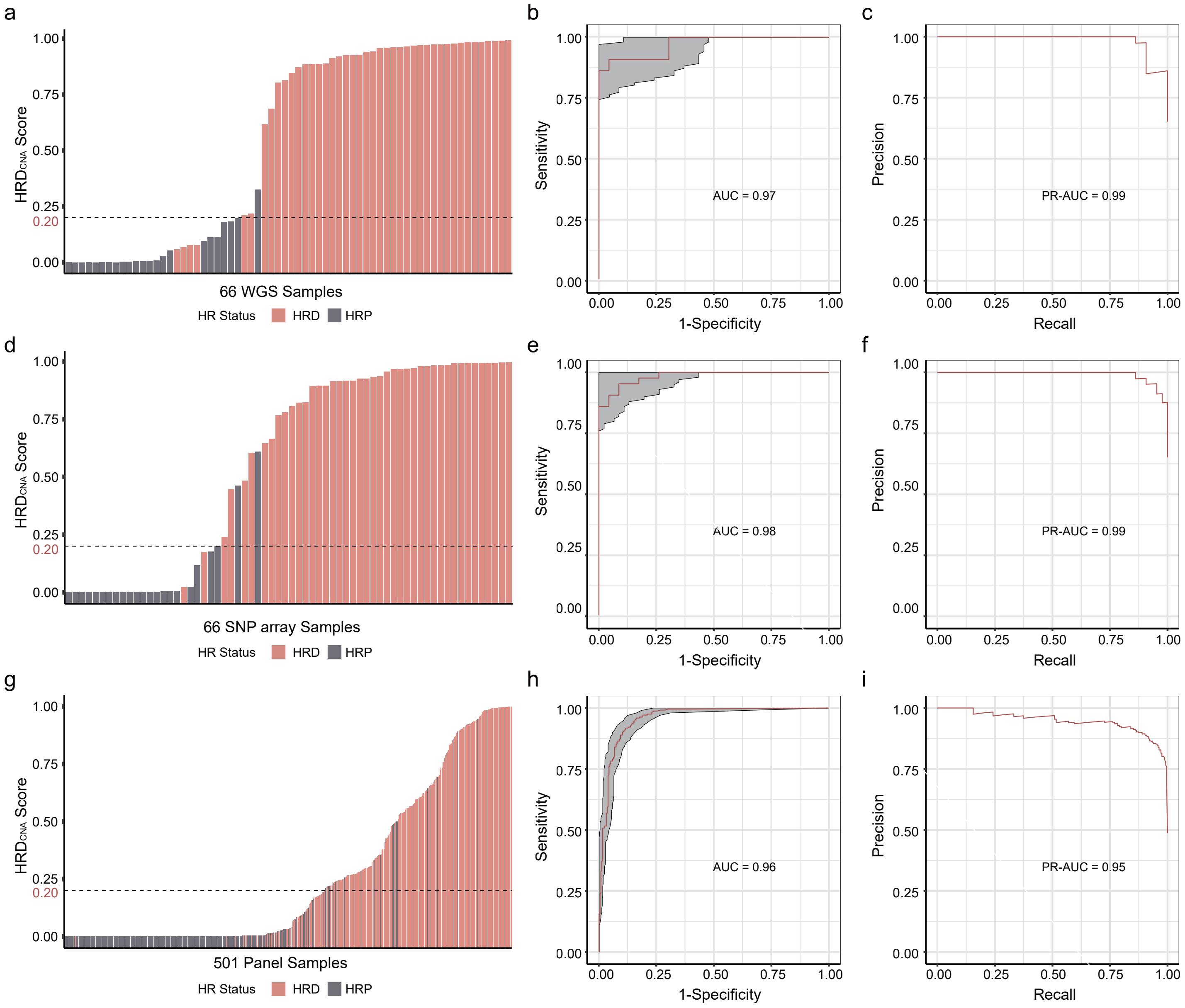

**Supplementary Fig. 3: Performance of $HRD_{CNA}$ model using data derived from different sequencing platforms.**

**a** $HRD_{CNA}$ scores for 66 breast WGS sequencing cancer samples are ordered from lowest to highest. The horizontal dashed line shows a cut-off score of 0.2. **b, c** ROC curve (**b**) and PR curve (**c**) showed the performance of $HRD_{CNA}$ model in 66 breast cancer samples with WGS sequencing. **d** $HRD_{CNA}$ scores for 66 breast SNP array sequencing cancer samples are ordered from lowest to highest. **e, f** ROC curve (**e**) and PR curve (**f**) showed the performance of $HRD_{CNA}$ model in 66 breast cancer samples with SNP array sequencing. **g** $HRD_{CNA}$ scores for 501 pan-cancer panel sequencing cancer samples are ordered from lowest to highest. **h, i** ROC curve (**h**) and PR curve (**i**) showed the performance of $HRD_{CNA}$ model in 501 pan-cancer cancer samples with panel sequencing. The gray shaded area represents a 95% confidence interval.

# Supplementary Figure 4

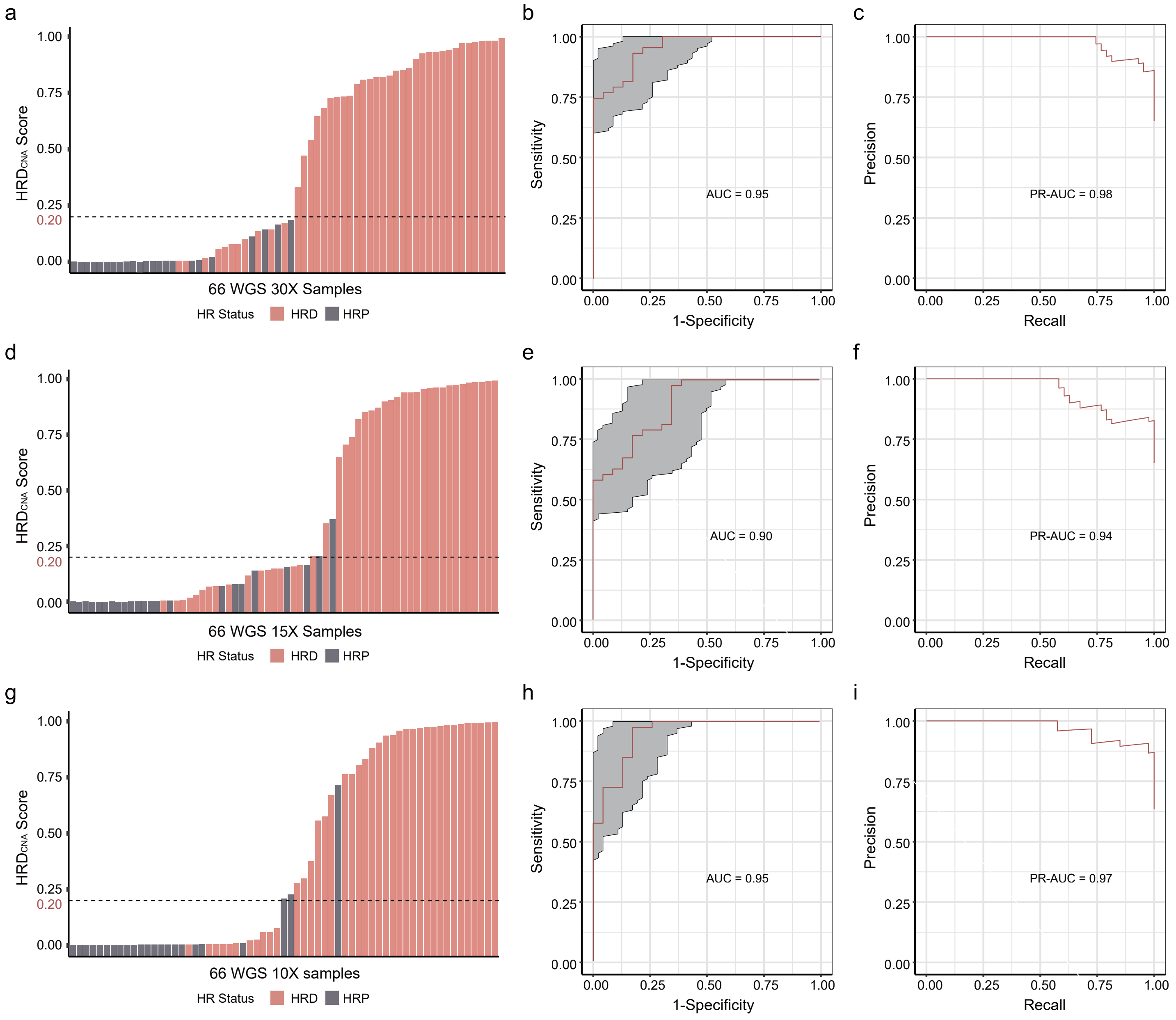

**Supplementary Fig. 4: Performance of $HRD_{CNA}$ model in WGS at different sequencing depths.**

**a** $HRD_{CNA}$ scores for 60 breast cancer WGS (30X) samples are ordered from lowest to highest. The horizontal dashed line shows a cut-off score of 0.2. **b, c** ROC curve (**b**) and PR curve (**c**) showed the performance of $HRD_{CNA}$ model in 66 breast cancer WGS (30X) samples. **d** $HRD_{CNA}$ scores for 66 breast cancer WGS (15X) samples are ordered from lowest to highest. **e, f** ROC curve (**e**) and PR curve (**f**) showed the performance of $HRD_{CNA}$ model in 66 breast cancer WGS (15X) samples. **g** $HRD_{CNA}$ scores for 66 breast cancer WGS (10X) samples are ordered from lowest to highest. **h, i** ROC curve (**h**) and PR curve (**i**) showed the performance of $HRD_{CNA}$ model in 66 breast cancer WGS (10X) samples. The gray shaded area represents a 95% confidence interval.

# Supplementary Figure 5

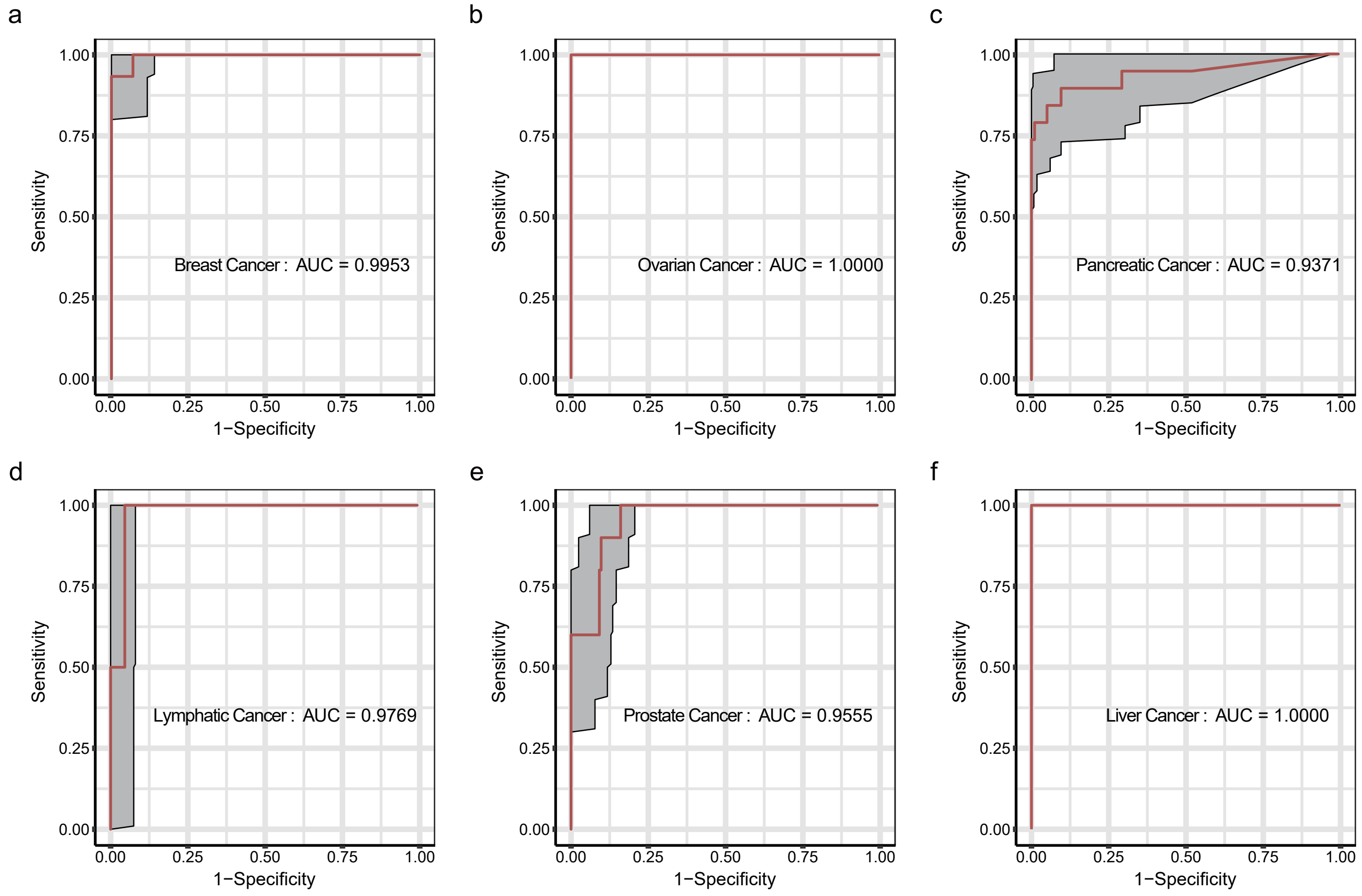

**Supplementary Fig. 5: Performance of $HRD_{CNA}$ model in individual cancer types.**

**a-f** ROC curve showed the performance of $HRD_{CNA}$ model in breast (**a**), ovarian (**b**), pancreatic (**c**), lymphatic (**d**), prostate (**e**), and liver (**f**) cancer samples. The gray shaded area represents a 95% confidence interval.

# Supplementary Figure 6

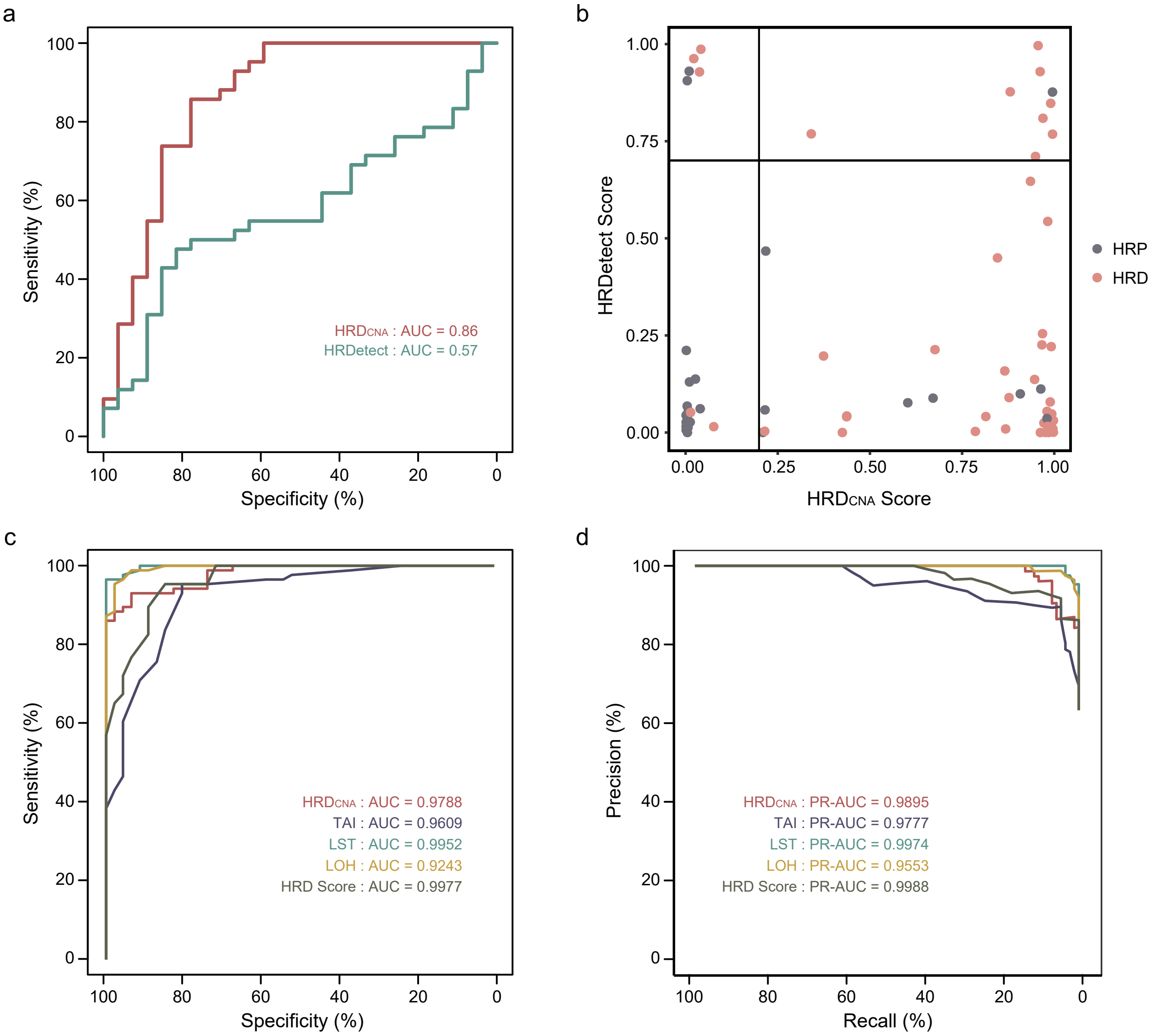

**Supplementary Fig. 6 Performance comparison of $HRD_{CNA}$ with HRD score and HRDetect.**

**a** ROC curves showed the performance of $HRD_{CNA}$ and HRDetect in HRD prediction in a cohort of 71 new TNBC patients in TCGA dataset. **b** The scatterplot displays the relationship between $HRD_{CNA}$ score and HRDetect score. **c, d** ROC curves (**c**) and PR curves (**d**) analysis showed the performance of $HRD_{CNA}$, LOH, TAI, LST, and HRD score in HRD prediction in two independent validation datasets: a total of 132 cancer samples, including 66 breast cancer samples with WGS and 66 breast cancer samples with SNP array sequencing. LOH, loss of heterozygosity; TAI, telomeric allelic imbalance; LST, large-scale transition.

# Supplementary Figure 7

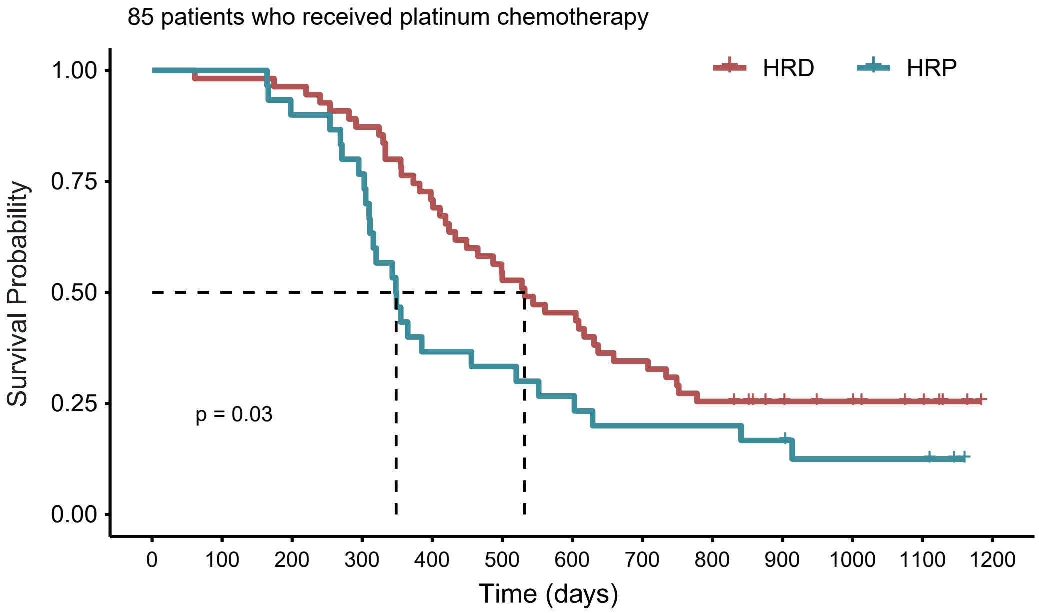

**Supplementary Fig. 7: HRD score in Kaplan-Meier (KM) survival analysis.** The KM survival analysis of HRD score in 85 patients who had received platinum chemotherapy from 501 pan-cancer cohort. *P* value is calculated using log-rank test.

# Supplementary Figure 8

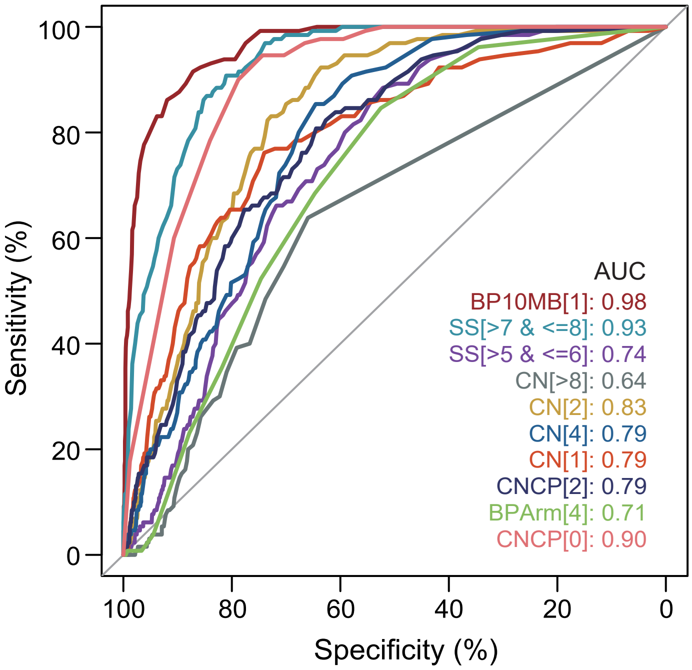

**Supplementary Fig. 8: Performance of individual CNA feature in HRD prediction.**

The performance of individual CNA feature in HRD prediction. BP10MB[1] or SS[>7 & <=8] show the top performance compared with the remaining CNA features.

# Supplementary Figure 9

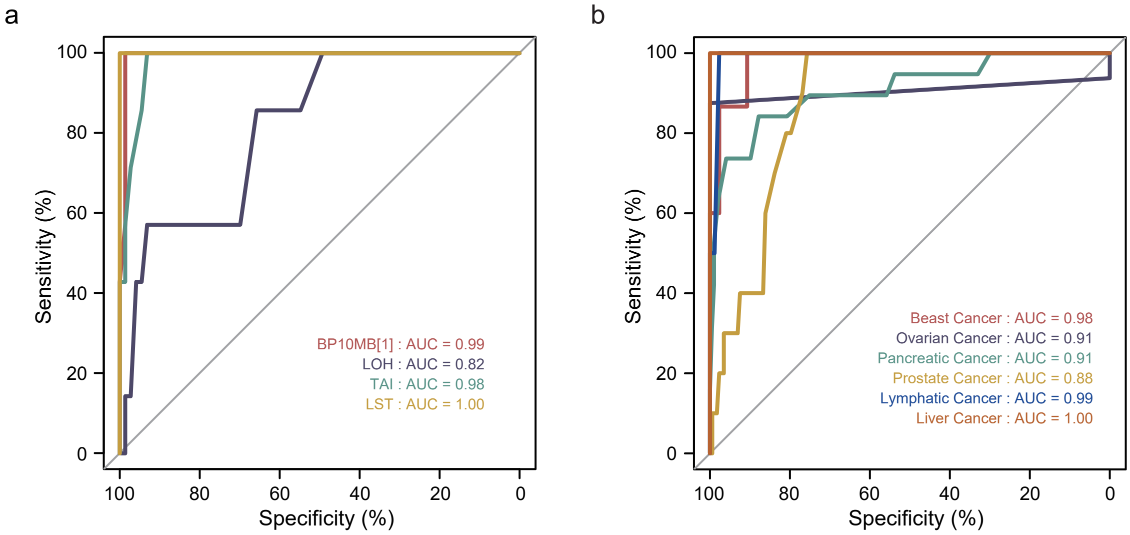

**Supplementary Fig. 9: Performance comparison between BP10MB[1] and other tools.**

**a** Comparison of the performance of BP10MB[1], LOH, TAI, and LST in HRD prediction in a cohort of 80 new breast cancers. **b** Performance of BP10MB[1] in individual cancer types. BP10MB[1], the number of breakpoints per 10MB of DNA is 1; LOH, loss of heterozygosity; TAI, telomeric allelic imbalance; LST, large-scale transition.

# Supplementary Figure 10

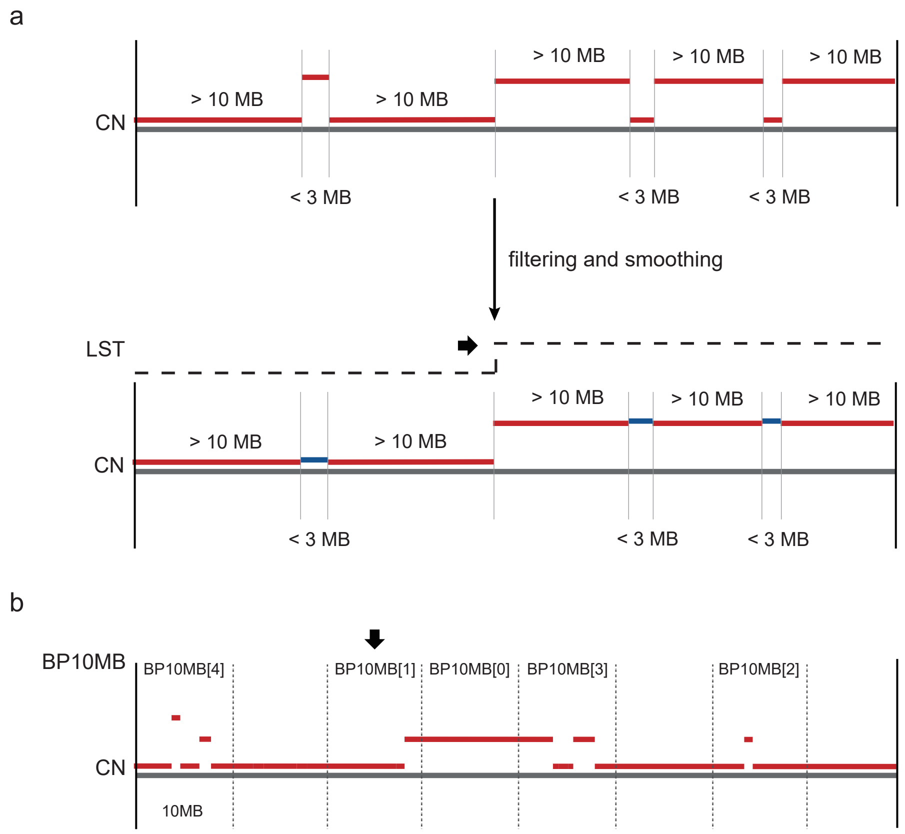

**Supplementary Fig. 10: Comparison between LST and BP10MB[1].**

**a** The example of LST detected. LST was defined as a chromosomal break between adjacent regions of at least 10 MB or larger, and the number of LST in the tumor genome was estimated for each chromosome arm independently (the centromeric breaks are not considered) and after filtering and smoothing of all variations less than 3 MB. The dashed line shows large-scale segments obtained after filtering and smoothing small-scale variations. CN, copy number; LST, large-scale transition. **b** The example of BP10MB[1] detected. BP10MB[1] indicates the breakpoint count per 10MB of DNA is 1. In the feature selection section before modeling, we also include the components BP10MB[0], BP10MB[2], BP10MB[3], BP10MB[4], BP10MB[5], and BP10MB[>5], which are classified from a feature BP10MB. They represent different counts of breakpoints per 10 MB, respectively (Supplementary Table 3).

## Supplementary Figure 11

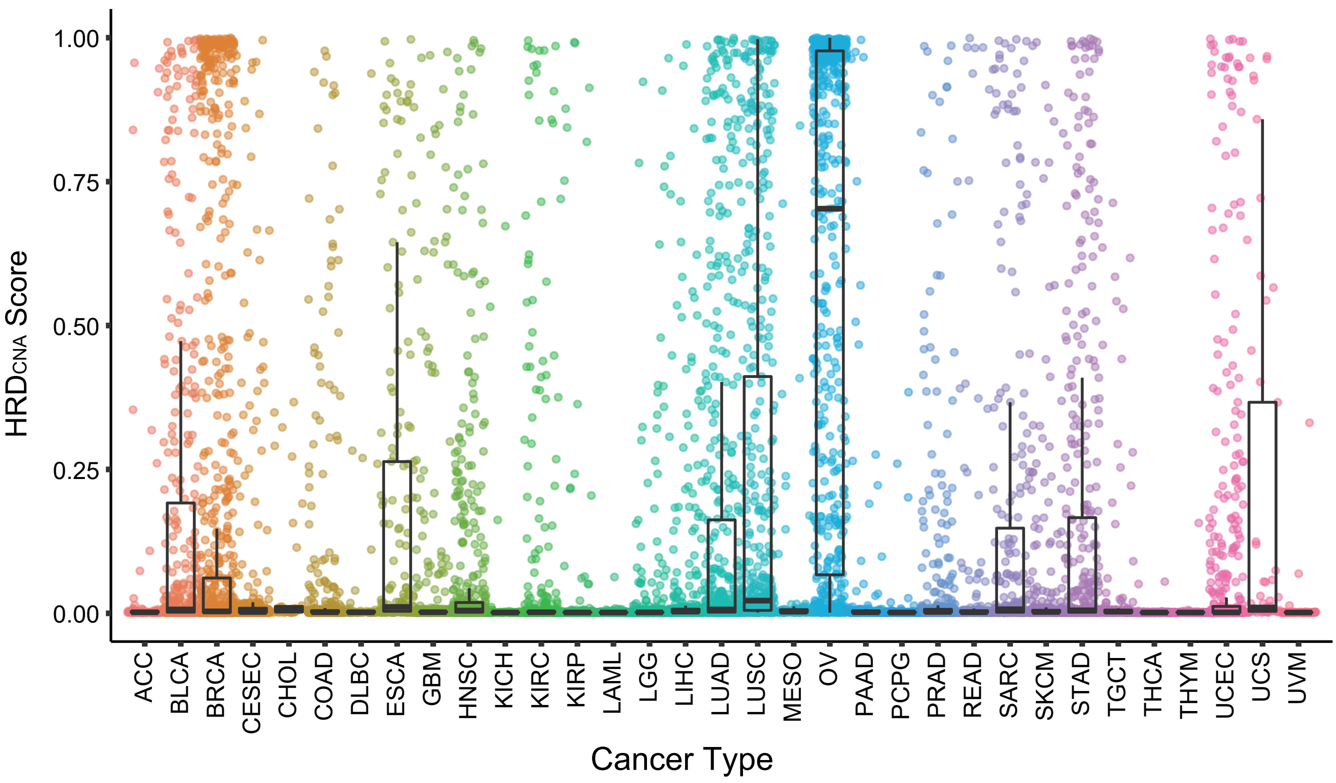

**Supplementary Fig. 11: Pan-cancer HRD landscape predicted using $HRD_{CNA}$.**

$HRD_{CNA}$ scores of 10,906 samples across 33 cancer types from the TCGA dataset. Each point represents a cancer sample.

Supplementary Figure 12

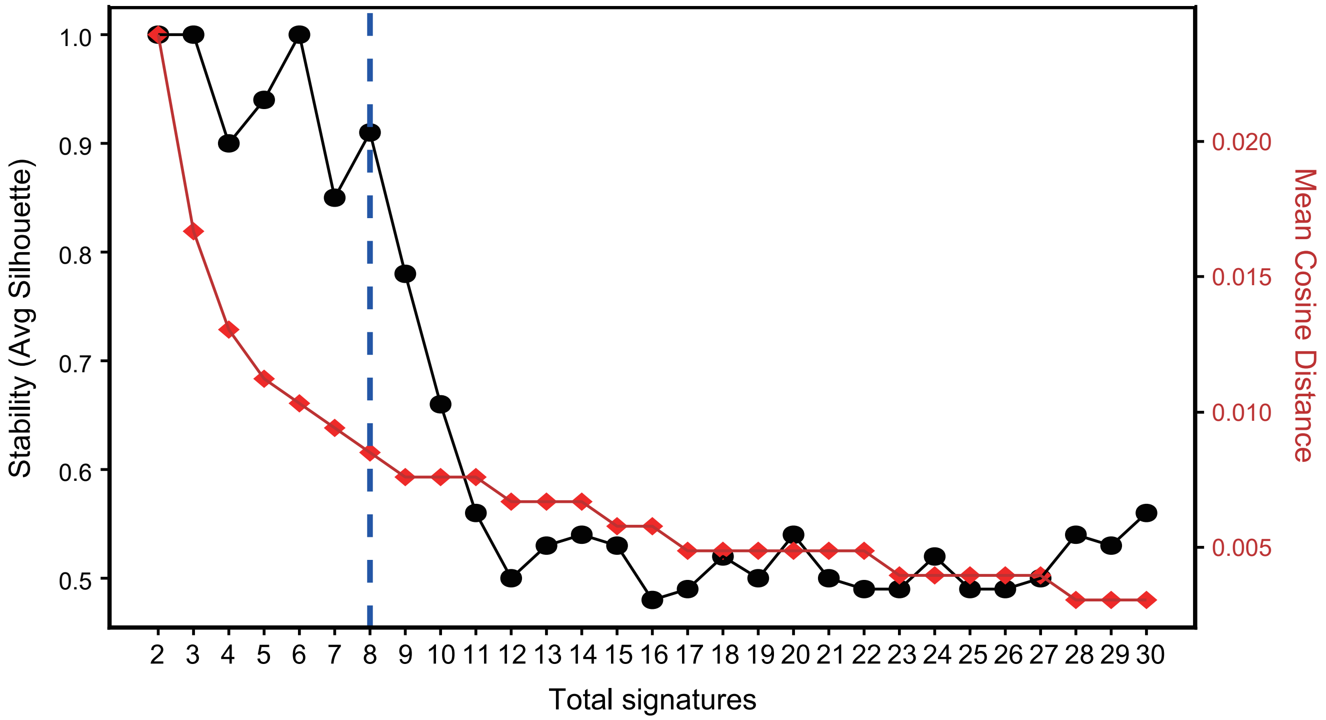

**Supplementary Fig. 12: Signature number determination for Sig-CNS model.**

8 signatures are selected due to their relatively high stability and low reconstruction error.

# Supplementary Figure 13

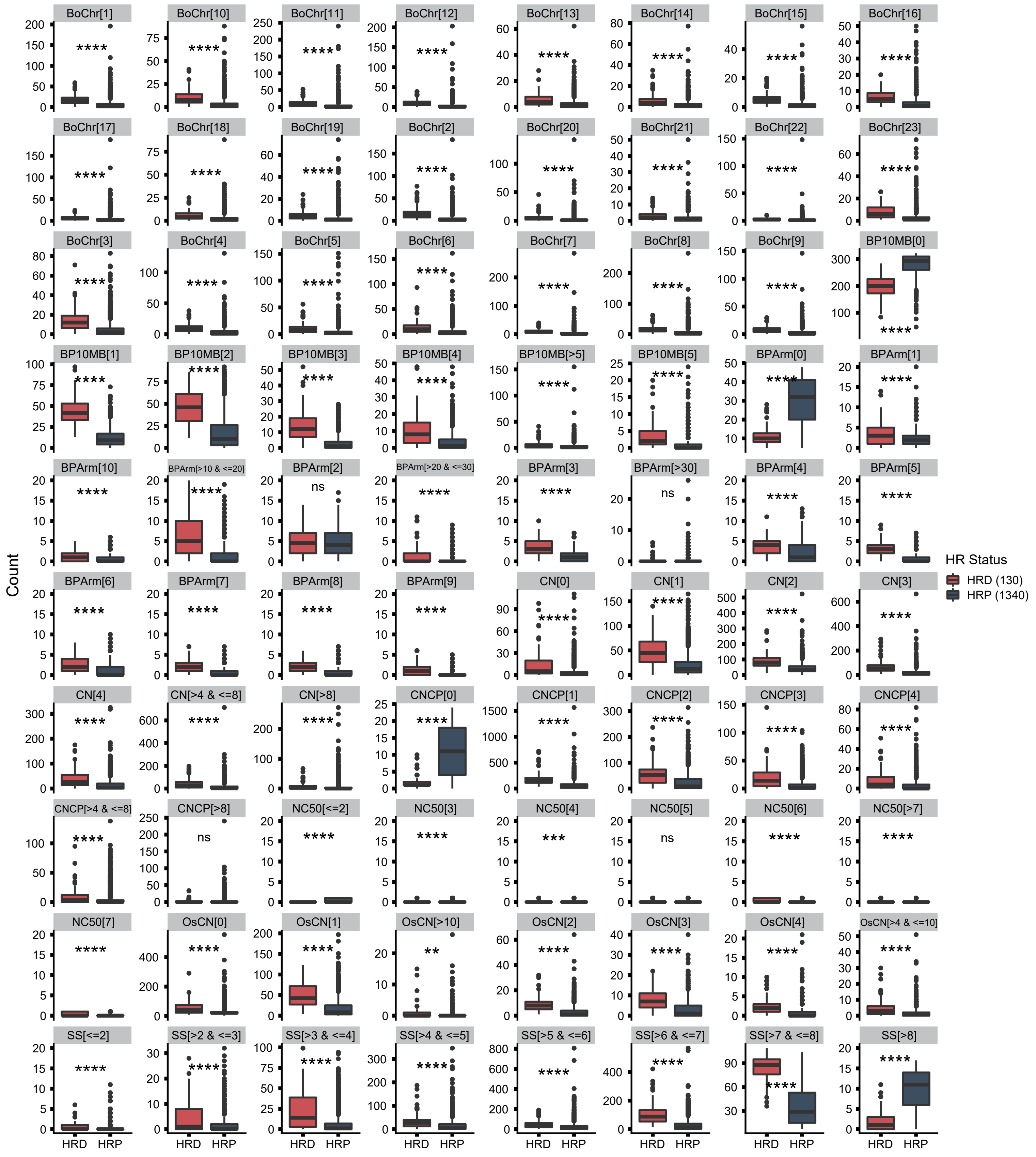

**Supplementary Fig. 13: The difference in 80 CNA features between HRD and HRP samples.**

Compare the difference in 80 CNA features between HRD and HRP samples. ****, $P < 0.0001$; ***, $P < 0.001$; **, $P < 0.01$; *, $P < 0.05$; ns, $P > 0.05$. *P* values are calculated using Wilcoxon test.

# Supplementary Figure 14

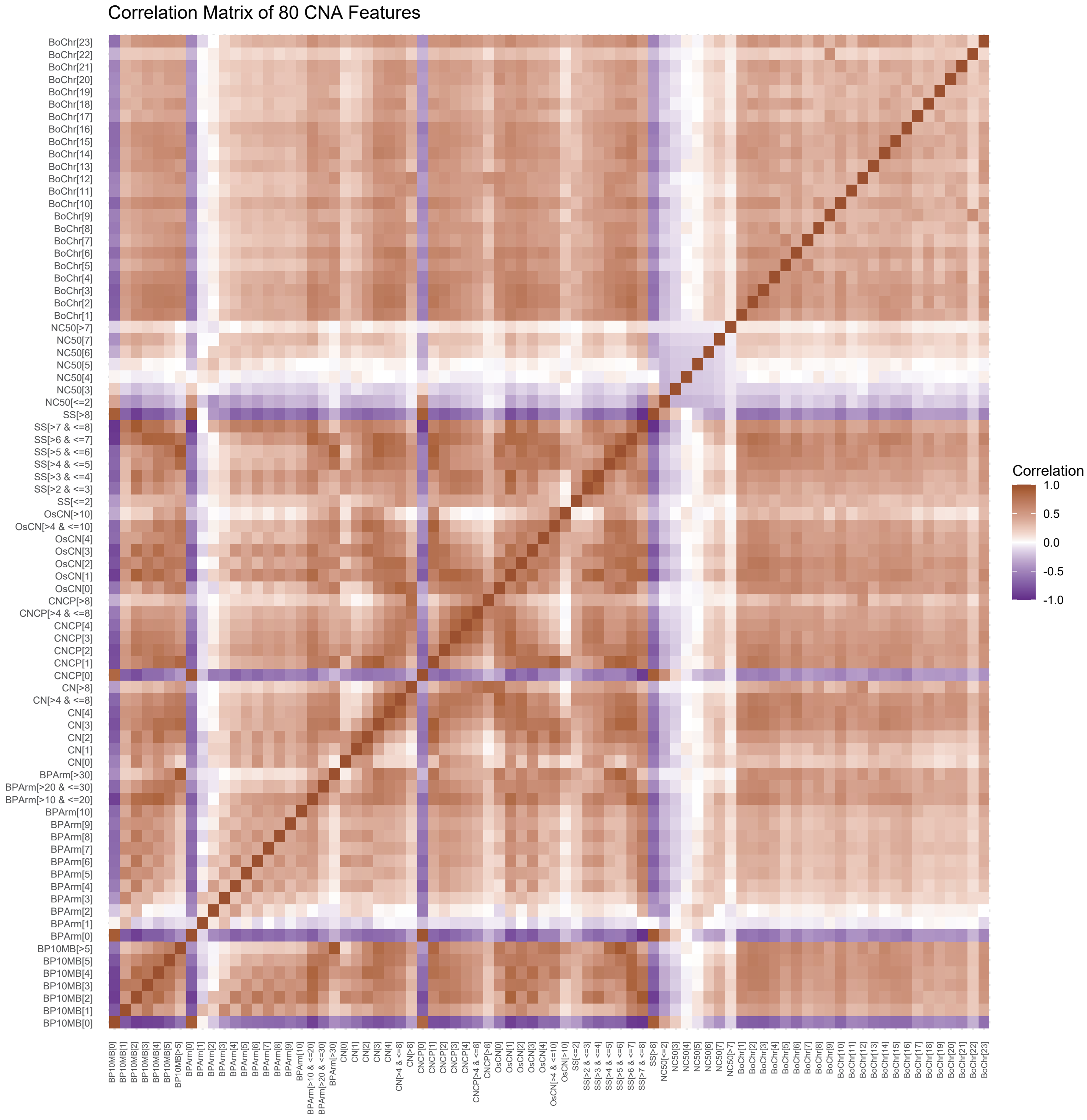

**Supplementary Fig. 14: The correlation matrix of 80 CNA features.**

The correlation matrix of 80 CNA features. Brown, positive correlation; purple, negative correlation. The depths of colors indicate the extent of the difference.

# Supplementary Figure 15

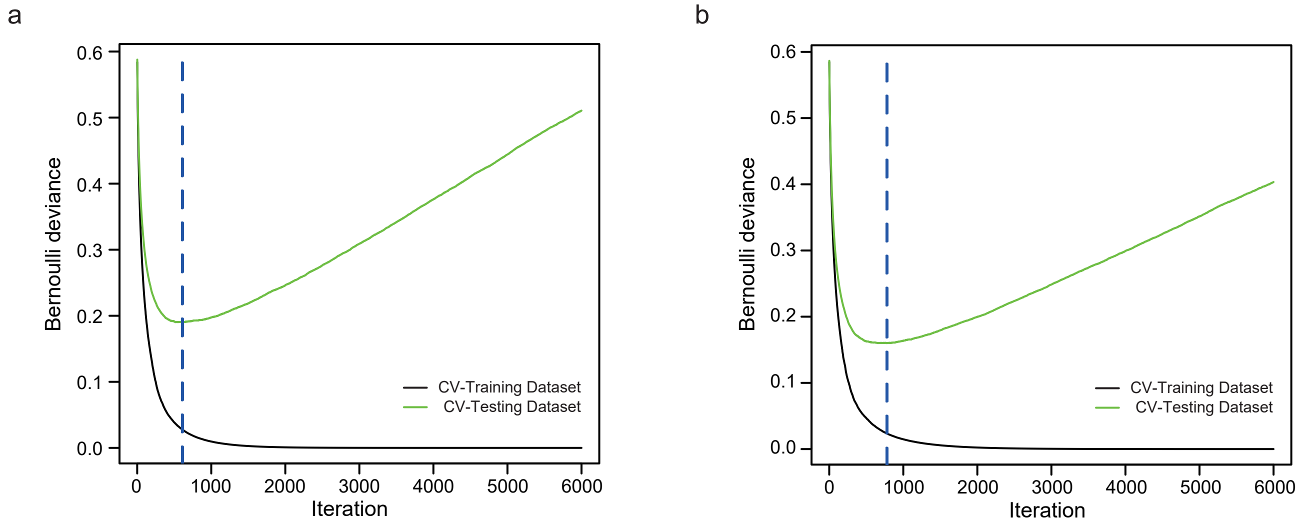

**Supplementary Fig. 15: Tree number selection for GBM models.**

Bernoulli deviance in cross-validation (CV) training (black) and testing (green) datasets as a function of iterations (number of trees). The blue dotted line indicated the best value that minimizes overfitting. **a** The model is trained using 76 CNA features that show significant differences between HRD and HRP groups, and the best number of trees is 572. **b** The model is trained using 10 CNA features with the top 10 relative influence score, and the best number of trees is 777.

**Supplementary Table 1: The predictive scores of variants of uncertain significance by different tools**

| HRDCNA Score | | 0.933088977 | 0.908444804 | 0.99087178 | 0.995341395 |
|---|---|---|---|---|---|
| Gene | | BRCA1 | BRCA2 | BRCA2 | BRCA2 |
| Nucleotide | | c.286G>C | c.1661G>T | c.2892A>T | c.4912A>G |
| Protein | | p.Asp96His | p.Cys554Phe | p.Lys964Asn | p.Lys1638Glu |
| CancerVar | Score | 10 | 7 | 5 | 7 |
| | Result | Pathogenic | Unknown | Unknown | Unknown |
| CADD | PHRED | 27.2 | 14.71 | 15.07 | 12.78 |
| Mutation Assessor | FI Score | 2.535 | 1.935 | 2.08 | 1.765 |
| | Func. Impact | medium | low | medium | low |
| PolyPhen-2 | HVarProb | 0.999 | 0.039 | 0.004 | 0.01 |
| | HVarPred | probably damaging | benign | benign | benign |
| | HDivProb | 1 | 0.159 | 0.009 | 0.012 |
| | HDivPred | probably damaging | benign | benign | benign |

**Supplementary Table 2: The cancer type frequencies in the training data for $HRD_{CNA}$**

| Cancer Type | Sample Number | Frequency |
|---:|---:|---:|
| Biliary | 8 | 0.005 |
| Bone/Soft tissue | 9 | 0.006 |
| Breast | 369 | 0.251 |
| Esophagus | 40 | 0.027 |
| Head and neck | 8 | 0.005 |
| Kidney | 66 | 0.045 |
| Liver | 196 | 0.133 |
| Lymphoid | 173 | 0.118 |
| Medulloblastoma | 109 | 0.074 |
| Myeloid | 21 | 0.014 |
| Ovary | 17 | 0.012 |
| Pancreas | 211 | 0.144 |
| Prostate | 179 | 0.122 |
| Skin | 48 | 0.033 |
| Stomach | 16 | 0.011 |

**Supplementary Table 3: The copy number features used for training HRD$_{CNA}$**

| Feature | Component | Label | Min | Max | Biological Meaning |
|---|---|---|---|---|---|
| BP10MB | BP10MB[0] | point | 0 | 0 | breakpoint count per 10 Mb |
| BP10MB | BP10MB[1] | point | 1 | 1 | |
| BP10MB | BP10MB[2] | point | 2 | 2 | |
| BP10MB | BP10MB[3] | point | 3 | 3 | |
| BP10MB | BP10MB[4] | point | 4 | 4 | |
| BP10MB | BP10MB[5] | point | 5 | 5 | |
| BP10MB | BP10MB[>5] | range | 5 | Inf | |
| BPArm | BPArm[0] | point | 0 | 0 | breakpoint count per chromosome arm |
| BPArm | BPArm[1] | point | 1 | 1 | |
| BPArm | BPArm[2] | point | 2 | 2 | |
| BPArm | BPArm[3] | point | 3 | 3 | |
| BPArm | BPArm[4] | point | 4 | 4 | |
| BPArm | BPArm[5] | point | 5 | 5 | |
| BPArm | BPArm[6] | point | 6 | 6 | |
| BPArm | BPArm[7] | point | 7 | 7 | |
| BPArm | BPArm[8] | point | 8 | 8 | |
| BPArm | BPArm[9] | point | 9 | 9 | |
| BPArm | BPArm[10] | point | 10 | 10 | |
| BPArm | Arm[>10 & <=20] | range | 10 | 20 | |
| BPArm | Arm[>20 & <=30] | range | 20 | 30 | |
| BPArm | BPArm[>30] | range | 30 | Inf | |
| CN | CN[0] | point | 0 | 0 | copy number of the segments |
| CN | CN[1] | point | 1 | 1 | |
| CN | CN[2] | point | 2 | 2 | |
| CN | CN[3] | point | 3 | 3 | |
| CN | CN[4] | point | 4 | 4 | |
| CN | CN[>4 & <=8] | range | 4 | 8 | |
| CN | CN[>8] | range | 8 | Inf | |
| CNCP | CNCP[0] | point | 0 | 0 | difference in copy number between adjacent segments |
| CNCP | CNCP[1] | point | 1 | 1 | |
| CNCP | CNCP[2] | point | 2 | 2 | |
| CNCP | CNCP[3] | point | 3 | 3 | |
| CNCP | CNCP[4] | point | 4 | 4 | |
| CNCP | CNCP[>4 & <=8] | range | 4 | 8 | |
| CNCP | CNCP[>8] | range | 8 | Inf | |
| OsCN | OsCN[0] | point | 0 | 0 | lengths of oscillating copy number segment chains |
| OsCN | OsCN[1] | point | 1 | 1 | |
| OsCN | OsCN[2] | point | 2 | 2 | |
| OsCN | OsCN[3] | point | 3 | 3 | |
| OsCN | OsCN[4] | point | 4 | 4 | |
| OsCN | sCN[>4 & <=10] | range | 4 | 10 | |
| OsCN | OsCN[>10] | range | 10 | Inf | |
| SS | SS[<=2] | range | -Inf | 2 | log10 based size of segments |
| SS | SS[>2 & <=3] | range | 2 | 3 | |
| SS | SS[>3 & <=4] | range | 3 | 4 | |
| SS | SS[>4 & <=5] | range | 4 | 5 | |
| SS | SS[>5 & <=6] | range | 5 | 6 | |
| SS | SS[>6 & <=7] | range | 6 | 7 | |
| SS | SS[>7 & <=8] | range | 7 | 8 | |
| SS | SS[>8] | range | 8 | Inf | |

| Feature | Component | Label | Min | Max | Biological Meaning |
|---|---|---|---|---|---|
| NC50 | NC50[<=2] | range | -Inf | 2 | minimal number of chromosome with 50% copy number variation |
| NC50 | NC50[3] | point | 3 | 3 | |
| NC50 | NC50[4] | point | 4 | 4 | |
| NC50 | NC50[5] | point | 5 | 5 | |
| NC50 | NC50[6] | point | 6 | 6 | |
| NC50 | NC50[7] | point | 7 | 7 | |
| NC50 | NC50[>7] | range | 7 | Inf | |
| BoChr | BoChr[1] | point | 1 | 1 | burden of chromosome |
| BoChr | BoChr[2] | point | 2 | 2 | |
| BoChr | BoChr[3] | point | 3 | 3 | |
| BoChr | BoChr[4] | point | 4 | 4 | |
| BoChr | BoChr[5] | point | 5 | 5 | |
| BoChr | BoChr[6] | point | 6 | 6 | |
| BoChr | BoChr[7] | point | 7 | 7 | |
| BoChr | BoChr[8] | point | 8 | 8 | |
| BoChr | BoChr[9] | point | 9 | 9 | |
| BoChr | BoChr[10] | point | 10 | 10 | |
| BoChr | BoChr[11] | point | 11 | 11 | |
| BoChr | BoChr[12] | point | 12 | 12 | |
| BoChr | BoChr[13] | point | 13 | 13 | |
| BoChr | BoChr[14] | point | 14 | 14 | |
| BoChr | BoChr[15] | point | 15 | 15 | |
| BoChr | BoChr[16] | point | 16 | 16 | |
| BoChr | BoChr[17] | point | 17 | 17 | |
| BoChr | BoChr[18] | point | 18 | 18 | |
| BoChr | BoChr[19] | point | 19 | 19 | |
| BoChr | BoChr[20] | point | 20 | 20 | |
| BoChr | BoChr[21] | point | 21 | 21 | |
| BoChr | BoChr[22] | point | 22 | 22 | |
| BoChr | BoChr[23] | point | 23 | 23 | |

**Supplementary Table 4: Sources of publicly available data**

| Data | | Source |
|---|---|---|
| PCAWG Dataset | Copy number profiles | https://dcc.icgc.org/releases/PCAWG/ |
| | BRCA1/2 status | https://www.nature.com/articles/s41467-020-19406-4 |
| 560 breast Dataset | Copy number profiles | http://medgen.medschl.cam.ac.uk/serena-nik-zainal/ |
| | BRCA1/2 status | https://www.nature.com/articles/nm.4292 |
| | Mutation data | https://www.nature.com/articles/nm.4292 |
| Panel Dataset | Copy number profiles | https://bmccancer.biomedcentral.com/articles/10.1186/s12885-022-09602-4 |
| | HR status | https://bmccancer.biomedcentral.com/articles/10.1186/s12885-022-09602-4 |
| | Survival data | https://bmccancer.biomedcentral.com/articles/10.1186/s12885-022-09602-4 |
| 66 breast Dataset | SNP array: Copy number profiles | https://doi.org/10.6084/m9.figshare.9808496 |
| | WGS: Copy number profiles | https://doi.org/10.6084/m9.figshare.9808505 |
| | WGS(30X): Copy number profiles | https://doi.org/10.6084/m9.figshare.9808511 |
| | WGS(15X): Copy number profiles | https://doi.org/10.6084/m9.figshare.9808514 |
| | WGS(10X): Copy number profiles | https://doi.org/10.6084/m9.figshare.9808517 |
| TCGA dataset | Copy number profiles | https://portal.gdc.cancer.gov/ |
| | Biallelic mutations in HR-related genes | https://www.nature.com/articles/s41467-017-00921-w |
| | Mutation data | https://gdc.cancer.gov/ |
| Pathogenicity annotations | | https://www.ncbi.nlm.nih.gov/clinvar/ |
