# Supplementary material for: Copy number alteration features in pan-cancer homologous recombination deficiency prediction and biology
Source: Commun Biol. 2023 May 16;6:527. doi: 10.1038/s42003-023-04901-3 (PMC10188435; doi:10.1038/s42003-023-04901-3)
Supplement: Supplementary file 2 — Reporting Summary [file 42003_2023_4901_MOESM2_ESM.pdf]

nature portfolio

Corresponding author(s): Xue-Song Liu

Last updated by author(s): Apr 23, 2023

Reporting Summary

Nature Portfolio wishes to improve the reproducibility of the work that we publish. This form provides structure for consistency and transparency in reporting. For further information on Nature Portfolio policies, see our Editorial Policies and the Editorial Policy Checklist.

Statistics

For all statistical analyses, confirm that the following items are present in the figure legend, table legend, main text, or Methods section.

n/a	Confirmed	
☐	☒	The exact sample size (n) for each experimental group/condition, given as a discrete number and unit of measurement
☐	☒	A statement on whether measurements were taken from distinct samples or whether the same sample was measured repeatedly
☐	☒	The statistical test(s) used AND whether they are one- or two-sided<br>*Only common tests should be described solely by name; describe more complex techniques in the Methods section.*
☐	☒	A description of all covariates tested
☐	☒	A description of any assumptions or corrections, such as tests of normality and adjustment for multiple comparisons
☐	☒	A full description of the statistical parameters including central tendency (e.g. means) or other basic estimates (e.g. regression coefficient) AND variation (e.g. standard deviation) or associated estimates of uncertainty (e.g. confidence intervals)
☐	☒	For null hypothesis testing, the test statistic (e.g. F, t, r) with confidence intervals, effect sizes, degrees of freedom and P value noted<br>*Give P values as exact values whenever suitable.*
☒	☐	For Bayesian analysis, information on the choice of priors and Markov chain Monte Carlo settings
☒	☐	For hierarchical and complex designs, identification of the appropriate level for tests and full reporting of outcomes
☒	☐	Estimates of effect sizes (e.g. Cohen's d, Pearson's r), indicating how they were calculated

Our web collection on statistics for biologists contains articles on many of the points above.

Software and code

Policy information about availability of computer code

Data collection

Somatic copy number data for the international cancer genome consortium (ICGC) portion of PCAWG dataset was downloaded at https://dcc.icgc.org/releases/PCAWG/consensus_cnv. BRCA1/2 status annotations for this dataset are obtained from the supplementary data in Nguyen et al. study (https://www.nature.com/articles/s41467-020-19406-4). Somatic copy number data of the 560 breast dataset were downloaded from the department of medical genetics at the University of Cambridge (http://medgen.medschl.cam.ac.uk/serena-nik-zainal/). BRCA1/2 status annotations and mutation data for this dataset are obtained from the supplementary data in Davies et al. study (https://www.nature.com/articles/nm.4292). Segment copy number variant and HR status information for the panel dataset is obtained from the supplementary data in Wen H et al. study (https://bmccancer.biomedcentral.com/articles/10.1186/s12885-022-09602-4). 66 breast dataset are publicly available in the figshare repository provided by de Luca et al. study (https://www.nature.com/articles/s41523-020-0172-0). Copy number data from ASCAT (array data) are available from https://doi.org/10.6084/m9.figshare.9808496 and ascatNGS (for original (https://doi.org/10.6084/m9.figshare.9808505) and downsampled WGS data are available from (30X: https://doi.org/10.6084/m9.figshare.9808511, 15X: https://doi.org/10.6084/m9.figshare.9808514, 10X: https://doi.org/10.6084/m9.figshare.9808517)). Somatic copy number data of TCGA pan-cancer dataset were downloaded from genomic data commons data portal (https://portal.gdc.cancer.gov/). Allele-specific copy number analysis of tumors was performed using ASCAT2, to generate integral allele-specific copy number profiles for the tumor cells. Biallelic inactivation in HR-related genes information for TCGA dataset are obtained from Riaz et al. study (https://www.nature.com/articles/s41467-017-00921-w). The mutation data from TCGA were obtained from the GDC Data Portal and Legacy Archive (https://gdc.cancer.gov/). The information on the cohort of 71 TNBC patients in TCGA dataset is derived from the supplementary data in Liao et al. study (https://bmcmedicine.biomedcentral.com/articles/10.1186/s12916-021-02068-4). The information on the cohort of 80 breast patients is derived from the supplementary data in Davies et al. study (https://www.nature.com/articles/nm.4292). Pathogenicity annotations were obtained from ClinVar (https://www.ncbi.nlm.nih.gov/clinvar/).

Data analysis	All code required to reproduce the analysis outlined in this manuscript are freely available at https://github.com/XSLiuLab/InterpretationAnalysisHRDCNA. Analyses can be read online at https://github.com/XSLiuLab/InterpretationAnalysisHRDCNA.

For manuscripts utilizing custom algorithms or software that are central to the research but not yet described in published literature, software must be made available to editors and reviewers. We strongly encourage code deposition in a community repository (e.g. GitHub). See the Nature Portfolio guidelines for submitting code & software for further information.

Data

Policy information about availability of data

All manuscripts must include a data availability statement. This statement should provide the following information, where applicable:

- Accession codes, unique identifiers, or web links for publicly available datasets
- A description of any restrictions on data availability
- For clinical datasets or third party data, please ensure that the statement adheres to our policy

All publicly available data sets included in this study are described in the "Data collection" section. Somatic copy number data for the international cancer genome consortium (ICGC) portion of PCAWG dataset was downloaded at https://dcc.icgc.org/releases/PCAWG/consensus_cnv. BRCA1/2 status annotations for this dataset are obtained from the supplementary data in Nguyen et al. study (https://www.nature.com/articles/s41467-020-19406-4). Somatic copy number data of the 560 breast dataset were downloaded from the department of medical genetics at the University of Cambridge (http://medgen.medschl.cam.ac.uk/serena-nik-zainal/). BRCA1/2 status annotations and mutation data for this dataset are obtained from the supplementary data in Davies et al. study (https://www.nature.com/articles/nm.4292). Segment copy number variant and HR status information for the panel dataset is obtained from the supplementary data in Wen H et al. study (https://bmccancer.biomedcentral.com/articles/10.1186/s12885-022-09602-4). 66 breast dataset are publicly available in the figshare repository provided by de Luca et al. study (https://www.nature.com/articles/s41523-020-0172-0). Copy number data from ASCAT (array data) are available from https://doi.org/10.6084/m9.figshare.9808496 and ascatNGS (for original (https://doi.org/10.6084/m9.figshare.9808505) and downsampled WGS data are available from (30X: https://doi.org/10.6084/m9.figshare.9808511, 15X: https://doi.org/10.6084/m9.figshare.9808514, 10X: https://doi.org/10.6084/m9.figshare.9808517)). Somatic copy number data of TCGA pan-cancer dataset were downloaded from genomic data commons data portal (https://portal.gdc.cancer.gov/). Allele-specific copy number analysis of tumors was performed using ASCAT2, to generate integral allele-specific copy number profiles for the tumor cells. Biallelic inactivation in HR-related genes information for TCGA dataset are obtained from Riaz et al. study (https://www.nature.com/articles/s41467-017-00921-w). The mutation data from TCGA were obtained from the GDC Data Portal and Legacy Archive (https://gdc.cancer.gov/). The information on the cohort of 71 TNBC patients in TCGA dataset is derived from the supplementary data in Liao et al. study (https://bmcmedicine.biomedcentral.com/articles/10.1186/s12916-021-02068-4). The information on the cohort of 80 breast patients is derived from the supplementary data in Davies et al. study (https://www.nature.com/articles/nm.4292). Pathogenicity annotations were obtained from ClinVar (https://www.ncbi.nlm.nih.gov/clinvar/). All derived data, and the raw data associated with the Figures of this study are freely available at https://github.com/XSLiuLab/InterpretationAnalysisHRDCNA. R markdown analysis report can be read online at https://github.com/XSLiuLab/InterpretationAnalysisHRDCNA.

Field-specific reporting

Please select the one below that is the best fit for your research. If you are not sure, read the appropriate sections before making your selection.

☒ Life sciences ☐ Behavioural & social sciences ☐ Ecological, evolutionary & environmental sciences

For a reference copy of the document with all sections, see nature.com/documents/nr-reporting-summary-flat.pdf

Life sciences study design

All studies must disclose on these points even when the disclosure is negative.

Sample size	No sample-size calculation was performed. We analysed all the suitable data (as according to exclusion criteria below) available to us. We collected 1,854 samples (WGS data) from PCAWG and 560 breast cancer samples (SNP array data). In total, 130 cancer samples with loss-of-function mutations in BRCA1/2 were labeled as HRD, while 1340 samples without inactivation mutations in known HR genes were labeled as HRP.
Data exclusions	To obtain a high-confidence training dataset of HRD, samples with BRCA1/2 deficiency were screened for classifier training. The selection criteria for BRCA1/2 deficient samples is to choose the samples with one of the following events in BRCA1/2: (i) complete copy number loss, (ii) LOH in combination with a pathogenic germline or somatic SNV/indel or structural variations, or (iii) pathogenic SNV/indels or structural variations in both alleles.
Replication	No primary experiments were carried out in this study. No biological or technical replicates were considered.
Randomization	No randomisation was performed in the study. All samples meeting the preset criteria were considered.
Blinding	Blinding was not relevant due to only bioinformatic analysis carried out in the study.

Reporting for specific materials, systems and methods

We require information from authors about some types of materials, experimental systems and methods used in many studies. Here, indicate whether each material, system or method listed is relevant to your study. If you are not sure if a list item applies to your research, read the appropriate section before selecting a response.

Materials & experimental systems

n/a	Involved in the study
☒	☐ Antibodies
☒	☐ Eukaryotic cell lines
☒	☐ Palaeontology and archaeology
☒	☐ Animals and other organisms
☒	☐ Human research participants
☒	☐ Clinical data
☒	☐ Dual use research of concern

Methods

n/a	Involved in the study
☒	☐ ChIP-seq
☒	☐ Flow cytometry
☒	☐ MRI-based neuroimaging
